# Life course socioeconomic position, alcohol drinking patterns in midlife, and cardiovascular mortality: Analysis of Norwegian population-based health surveys

**DOI:** 10.1371/journal.pmed.1002476

**Published:** 2018-01-02

**Authors:** Eirik Degerud, Inger Ariansen, Eivind Ystrom, Sidsel Graff-Iversen, Gudrun Høiseth, Jørg Mørland, George Davey Smith, Øyvind Næss

**Affiliations:** 1 Norwegian Institute of Public Health, Oslo, Norway; 2 Department of Psychology, University of Oslo, Oslo, Norway; 3 School of Pharmacy, University of Oslo, Oslo, Norway; 4 Diakonhjemmet Hospital, Center for Psychopharmacology, Oslo, Norway; 5 Institute of Clinical Medicine, University of Oslo, Oslo, Norway; 6 MRC Integrative Epidemiology Unit, School of Social and Community Medicine, University of Bristol, Bristol, United Kingdom; 7 Institute of Health and Society, University of Oslo, Oslo, Norway; University of Toronto, CANADA

## Abstract

**Background:**

Socioeconomically disadvantaged groups tend to experience more harm from the same level of exposure to alcohol as advantaged groups. Alcohol has multiple biological effects on the cardiovascular system, both potentially harmful and protective. We investigated whether the diverging relationships between alcohol drinking patterns and cardiovascular disease (CVD) mortality differed by life course socioeconomic position (SEP).

**Methods and findings:**

From 3 cohorts (the Counties Studies, the Cohort of Norway, and the Age 40 Program, 1987–2003) containing data from population-based cardiovascular health surveys in Norway, we included participants with self-reported information on alcohol consumption frequency (*n* = 207,394) and binge drinking episodes (≥5 units per occasion, *n* = 32,616). We also used data from national registries obtained by linkage. Hazard ratio (HR) with 95% confidence intervals (CIs) for CVD mortality was estimated using Cox models, including alcohol, life course SEP, age, gender, smoking, physical activity, body mass index (BMI), systolic blood pressure, heart rate, triglycerides, diabetes, history of CVD, and family history of coronary heart disease (CHD). Analyses were performed in the overall sample and stratified by high, middle, and low strata of life course SEP. A total of 8,435 CVD deaths occurred during the mean 17 years of follow-up. Compared to infrequent consumption (<once/month), moderately frequent consumption (2–3 times per week) was associated with a lower risk of CVD mortality (HR = 0.78, 95% CI 0.72, 0.84) overall. HRs for the high, middle, and low strata of SEP were 0.66 (95% CI 0.58, 0.76), 0.87 (95% CI 0.78, 0.97), and 0.79 (95% CI 0.64, 0.98), respectively, compared with infrequent users in each stratum. HRs for effect modification were 1.30 (95% CI 1.10, 1.54, *p* = 0.002; middle versus high), 1.23 (95% CI 0.96, 1.58, *p* = 0.10; low versus high), and 0.96 (95% CI 0.76, 1.21, *p* = 0.73; low versus middle). In the group with data on binge drinking, 2,284 deaths (15 years) from CVDs occurred. In comparison to consumers who did not binge during the past year, HRs among frequent bingers (≥1 time per week) were 1.58 (95% CI 1.31, 1.91) overall, and 1.22 (95% CI 0.84, 1.76), 1.71 (95% CI 1.31, 2.23), and 1.85 (95% CI 1.16, 2.94) in the strata, respectively. HRs for effect modification were 1.36 (95% CI 0.87, 2.13, *p* = 0.18; middle versus high), 1.63 (95% CI 0.92, 2.91, *p* = 0.10; low versus high), and 1.32 (95% CI 0.79, 2.20, *p* = 0.29; low versus middle). A limitation of this study was the use of a single measurement to reflect lifetime alcohol consumption.

**Conclusions:**

Moderately frequent consumers had a lower risk of CVD mortality compared with infrequent consumers, and we observed that this association was more pronounced among participants with higher SEP throughout their life course. Frequent binge drinking was associated with a higher risk of CVD mortality, but it was more uncertain whether the risk differed by life course SEP. It is unclear if these findings reflect differential confounding of alcohol consumption with health-protective or damaging exposures, or differing effects of alcohol on health across socioeconomic groups.

## Abstract 2

In an analysis of population-based health surveys from Norway, Eirik Degerud and colleagues examine the associations between life course socioeconomic position, alcohol drinking patterns in midlife, and cardiovascular mortality.

## Author summary

**Why was this study done?:**

- Individuals with low socioeconomic position tend to consume alcohol less frequently than individuals with middle or high socioeconomic position but experience more alcohol-related hospitalisations and deaths.
- The study was performed to assess whether the relation between drinking patterns and cardiovascular disease differs by socioeconomic position.

**What did the researchers do and find?:**

- We obtained information on socioeconomic factors throughout the life course of Norwegian adults and categorised them into low, middle, or high position.
- We found that moderately frequent alcohol consumers had a lower risk of dying from cardiovascular disease than infrequent consumers, and that this was more pronounced among those with high position. Very frequent consumption was associated with increased risk of CVD mortality, but only among those with low socioeconomic position.
- We also found that weekly binge drinkers had higher risk of dying from cardiovascular disease than current drinkers who did not binge drink the past year, but we could not elucidate whether the risk differed by life course socioeconomic position.

**What do these findings mean?:**

- The study observed socioeconomic differences in risk estimates of CVD mortality associated with given alcohol consumption levels. It is unclear if this reflects differential confounding of alcohol consumption with health-protective or damaging exposures, or differing effects of alcohol on health across socioeconomic groups.
- The heterogeneity between groups in the population needs to be assessed when making population recommendations regarding alcohol consumption.

## Introduction

Socioeconomic position (SEP) is relevant to behaviours, exposures, and susceptibilities that may influence health [1], such as social support, financial resources, or the knowledge, awareness, and determination required to actively follow a healthy lifestyle or consult a physician if needed. There is an inverse socioeconomic gradient in the exposure to risk factors for cardiovascular diseases (CVDs) [2], which translates into a gradient in the risk of clinical CVD events [3,4]. The majority of heart attacks and strokes occur in late adulthood, but atherosclerosis development starts in childhood [5]. Socioeconomic disadvantage at different stages throughout the life course could therefore be relevant to risk factor exposure, atherosclerosis development, and the long-term risk of clinical cardiovascular events [6–10].

In contrast to tobacco smoking, which is more frequent among socioeconomically disadvantaged individuals and has a clear detrimental effect on health, the relationship between SEP, alcohol, and health is less clear. Disadvantaged groups tend to report less frequent alcohol consumption but experience more harm from a given level of alcohol exposure [11–14]. This is sometimes referred to as the alcohol harm paradox [15]. In terms of CVDs, associations between alcohol drinking patterns and CVD risk further complicate the situation. A drinking pattern characterised by more frequent consumption of low to moderate volumes is associated with a reduced risk in comparison to infrequent drinking or abstainers, while episodic heavy drinking, also called binge drinking, is associated with an increased risk [16]. Alcohol has multiple biological effects on the cardiovascular system, both harmful and potentially protective [17–20], and it has been suggested that differing dose-response relationships of these mechanisms may explain the overall J-shaped risk curve.

Biological effects of alcohol should not differ by SEP, but the noncausal associations could do so if the lifestyles that accompany a drinking pattern differ according to SEP [21]. When consuming alcohol, for example, disadvantaged individuals may more frequently co-consume junk food or smoke cigarettes, while advantaged individuals may be more prone to combine drinking with advantageous health-related behaviours and characteristics [21]. These potential differences may be profound and captured by the measurement of important risk factors but may also be subtle and difficult to measure individually. The assessment of SEP throughout the life course could be an approach that encapsulates the effect of these potentially subtle differences over time. In this study, we investigated whether the diverging relationships between alcohol drinking patterns and CVD mortality differed by life course SEP.

## Methods

### Study population

The Counties Studies [22], the Cohort of Norway [23], and the Age 40 Program [24] are three partly overlapping cohorts containing data from Norwegian population-based health surveys (1974–2003). Participants were recruited to the surveys through their personal identification number (PIN), which is unique to each inhabitant of Norway. The surveys assessed CVD risk factors, and a subset (1987–2003, *n* = 330,745) assessed the frequency of alcohol consumption. A further subsample also assessed the frequency of binge drinking episodes. The number of participants and age distribution in the surveys are provided (S1 Table).

### Data linkage

We linked data from the cohorts and national registries (the National Registry, the National Educational Database, and the Cause of Death Registry) by the use of PINs and a trusted third party (Statistics Norway). Data were sent from each source to the third party, which substituted the PIN with dummy numbers and sent the de-identified data to the authors. The authors then used the dummy numbers to link the data.

### Ethical approval and study protocol

This study is part of a larger research project. The data linkage and the research project was approved by the Regional Ethics Committee South-East (11/1676). The Ethics Committee also gave exemption regarding consent in older surveys in which consent was not obtained. The project protocol (S1 Protocol) as well as a description of differences from the protocol and the study performed (S1 Text) is included. This study is reported as per RECORD guidelines (S1 Checklist).

### Participants

Participants were eligible for the study if they were born before October 15, 1960, to 2 Norwegian parents, did not emigrate or die until after September 20, 1990, and if they completed the mandatory censuses in Norway from 1960 through 1990. These criteria were used to provide a sample that could be analysed with respect to life course SEP. Because of cohort overlap and individuals taking part in more than one survey, some participants were represented by multiple observations in the linked data. To optimise sample size, we selected 1 observation per participant, conditional on whether the observation had data on alcohol consumption frequency and placing priority on cohorts with longer follow-up. Eligible participants who had missing values on alcohol consumption frequency, CVD risk factors or indicators of SEP, or inconsistent follow-up data were excluded. The resulting sample was included in statistical analyses using the exposure variable alcohol consumption frequency. A subgroup of this sample was included in analyses of binge drinking episodes, which were available from some surveys.

### Alcohol exposure

The assessment of alcohol exposure differed between the source surveys, and we harmonised the data for use in the current study (S2 Table and S3 Table). Data identifying current and lifetime abstainers were harmonised into current abstainers for the main statistical analyses. Among current drinkers, alcohol consumption frequency was categorised into ‘Infrequent’, ‘Once per month to once per week’, ‘2–3 times per week’, and ‘4–7 times per week’. In surveys in which beer, wine, and liquor consumption were assessed separately, we first recoded the reported ordinal frequency categories into days of alcohol consumption per month, then summed the days to reflect total alcohol consumption, and finally recoded the sum back into the ordinal categories for harmonisation. This approach assumes that each beverage type was consumed on different days of the month. Participants reporting to be an abstainer on one question and reporting drinking on another question were defined as drinkers.

We defined a standard unit as 12.8 grams of pure alcohol, corresponding to a small bottle of beer (33.3 cl, 4.5%, 11.8 g), a glass of wine (15 cl, 12%, 14g), or a small glass or shot of liquor (4 cl, 40%, 12.6g). The frequency of heavy drinking episodes (5+ units or 60+ g of pure alcohol on a single occasion), which reflects the intake of high volumes, was categorised into ‘Not last year’, ‘A few times’, ‘1–3 times per month’, and ‘≥1 time per week’. The average amount of alcohol (g/day) could be assessed and harmonised for a subsample. Three calculations were applied, depending on which questions were available in each survey. Two calculations combined the average number of units consumed per occasion (0–20; higher values were truncated to 20) with the drinking frequency reported either per month (0–30) or in ordinal categories (4–7/week = 286/year, 2–3/week = 130/year, once/week = 52/year, 2–3/month = 30/year, once/month = 12/year, infrequent = 6/year). The third calculation was based on the total number of units consumed of beer, wine, and liquor in the course of 2 weeks.

### Life course SEP

CVDs tend to develop throughout the life course and manifest clinically in late adulthood. The manner in which risk factors and protective factors influence disease development may not be in unison; for example, there could be critical or sensitive time periods. A life course approach to epidemiology is one that takes this notion of time into account by acknowledging that measuring risk factors only once could be inadequate in order to assess the full impact they may have through the life course [25,26]. Previous studies have observed that CVD mortality is related to the number of occasions individuals have been exposed to socioeconomic disadvantage, measured by adding multiple indicators from different periods in the life course together in a cumulative manner [6,27].

We obtained a cumulative measure of life course SEP by combining indicators on household conditions from mandatory population and household censuses in 1960, 1970, and 1980 (type of dwelling, apartment block, row or detached house, ownership status, rooms per household capita, telephone ownership, access to water closet, and bath inside the dwelling), household income from the census in 1990, and the highest level of obtained education ever recorded until 2011 (National Educational Database). In contrast to the 1960 and 1970 censuses, which obtained almost complete population and household data, the census in 1980 did not pursue missing household data to the same extent. The household indicators have previously been observed to be independently associated with cause-specific mortality, as well as when combined into cumulative indexes [27,28]. A more detailed description of the role of the use of household indicators may be found here [1,25]. The household conditions, household income, and education provided a total of 20 indicators, which were scored (0 or 1) and given equal weight by summing the scores to construct the cumulative index (range 0–20). A high score indicated disadvantage and low life course SEP.

### Covariates and outcome

The health surveys provided self-reported data on current smoking, physical activity, diabetes, previous CVD (myocardial infarction, stroke, or angina pectoris), family history of coronary heart disease (CHD), objective measurements of blood pressure and heart rate, anthropometry, and biochemical nonfasting measurements (mmol/l) of serum triglycerides, total cholesterol, and high-density lipoprotein cholesterol (HDL-C). The Norwegian Cause of Death Registry provided outcome data on causes of death using the ninth and tenth revision of the International Classification of Diseases (ICD). The primary outcome was CVD mortality (1990–1995: ICD-9 390–459; 1996–2014: ICD-10: I00–I99). Three secondary outcomes were added in response to peer review, including death from ischemic heart disease (IHD) (1990–1995: ICD-9 410–414; 1996–2014: ICD–10: I20–I25), death from cerebrovascular diseases (1990–1995: ICD-9 430–438; 1996–2014: ICD-10: I60–I69), and all-cause mortality. The registry is almost exclusively based on certificates filled out by on-site medical doctors, and in the few cases in which autopsies are performed, 32% of deaths are reclassified over major ICD-10 chapters [29].

### Statistical analysis

We described the study population according to categories of life course SEP (index score 0–5 = high; 6–9 = middle; ≥10 = low) as well as according to alcohol consumption frequency within categories of SEP. Continuous variables were presented as mean (SD) and categorical variables as counts (%). Analysis of variance and chi-squared tests assessed differences between the groups. In survival analyses, we followed participants prospectively until emigration (December 31, 2012), death from any cause, or December 31, 2014. Cox Proportional Hazard Models estimated hazard ratios (HRs) and confidence intervals (CIs). Visual inspection of scaled Schoenfeld residuals against time did not indicate strong deviation from the assumption of proportional hazard. All analyses were conducted in R statistical software using *R studio* 1.0.44 [30], with additional use of the packages *survival* [31] and *mice* [32]. We did not impose a *p*-value cutoff to define statistical significance [33] nor apply survey weights.

To evaluate whether the SEP index was relevant to the outcome, we estimated the risk of CVD mortality in a model with the SEP index, age, and gender. Potential mediators, such as smoking, were not included, in order to assess the total effect. The index was first modelled using a smoothed penalised spline, allowing for a visual presentation of the functional relationship with the outcome, and then as a continuous and categorical variable for a formal assessment.

The aim was to assess if the relation between alcohol drinking patterns and the risk of CVD mortality differed by life course SEP. The hypothesis we tested statistically was whether SEP modified the effect of alcohol drinking patterns on the risk of CVD. We present HRs with 95% CI for alcohol consumption frequency and for the frequency of binge drinking episodes overall, in strata of SEP, and measures of effect modification on a multiplicative scale as HRs with 95% CI and *p*-values. Both exposures were modelled as ordinal categorical variables, with infrequent consumers and those who did not binge drink the last year as reference categories, respectively. Current abstainers were modelled separately as a dichotomous variable, with infrequent consumers as the reference category. Confounders of the relation between alcohol and CVD that were adjusted for included age, gender, current smoking, physical activity, body mass index (BMI), systolic blood pressure, heart rate, triglycerides, diabetes, history of CVD, and family history of CHD. In the subgroup with data on binge drinking, we adjusted the risk of CVD mortality according to alcohol consumption frequency for episodes of heavy drinking, and vice versa. Analyses were performed separately for total CVD, IHD, stroke, and all-cause mortality. Missing values were handled by list-wise deletion and totalled to 18.4%. We also performed missing value imputations of CVD risk factors and census data by chained equations among 245,336 eligible individuals with data on alcohol consumption (*n* = 38,284 with data on binge drinking). This reduced the amount of missing values to 3.5%. Alcohol exposure variables, CVD risk factors, census data, outcome data, and the SEP index were included in the imputation model and 10 data sets were generated. We then reanalysed the relationships with total CVD in each data set and report pooled HRs with 95% CIs.

We performed 2 sensitivity analyses in response to peer review. In the subgroup with data on binge drinking, we reanalysed the relation between alcohol consumption frequency and the risk of CVD while excluding binge drinkers (‘≥1 time per month’). In the subgroup with data on lifetime abstaining, we compared the risk of CVD when using lifetime abstainers and infrequent consumers as reference categories.

We added analyses while performing planned statistical analyses, which were elaborated during peer review. Short-term experimental studies show a dose-response relationship between alcohol intake and levels of HDL-C [17]. Using ordinary least squares regression and models adjusted for age and sex, we regressed HDL-C on a continuous variable of drinking frequency (4 categories of current drinkers). In a subsample, we also regressed HDL-C on a continuous variable of the average amount of alcohol consumed per day (g/day). Changes in HDL-C were compared to the dose-response relationship in a meta-analysis of experimental studies [17] to indicate if the main study variable was consistent with an increase in total alcohol consumption as judged by HDL-C and to indicate if the self-reported data were underreported. We also reanalysed the relationship between HDL-C and drinking frequency in strata of SEP to indicate if SEP could influence the ability to report consistently [34]. A formal test for a difference in slope was performed by including an interaction term between drinking frequency and SEP.

## Results

### Participants

**Fig 1:**
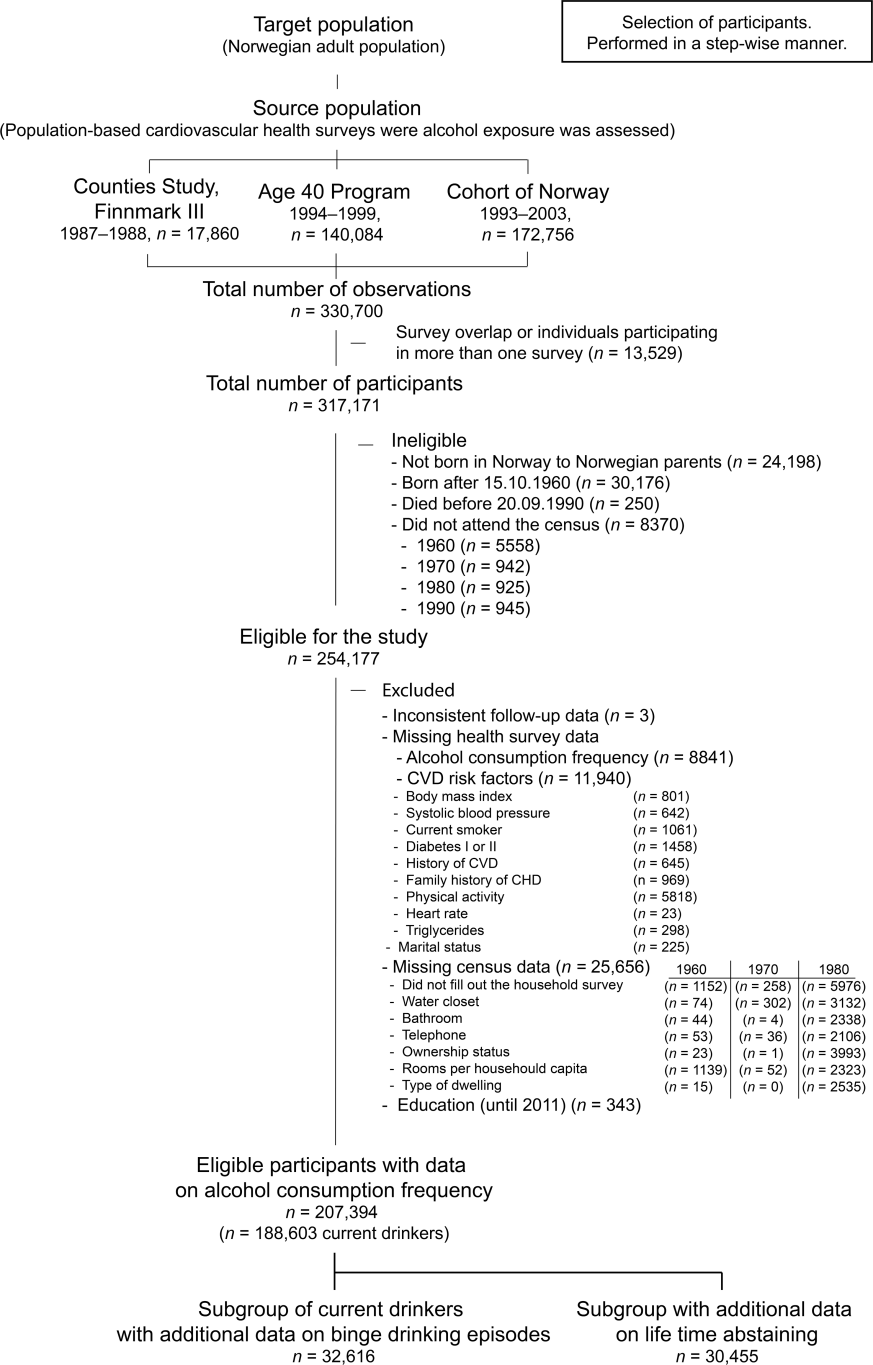
Flow chart showing inclusion and exclusion. CHD, coronary heart disease; CVD, cardiovascular disease.

From 330,700 potentially eligible observations, we selected (Fig 1) 1 observation per participant (*n* = 317,171). Participants with an immigration history (*n* = 24,198), who were born after October 15, 1960 (*n* = 30,176), or died before September 20,1990 (*n* = 250), or who did not attend one or more of the censuses (*n* = 8,370) were considered not eligible. We further excluded 18.4% of the 254,177 eligible participants for inconsistent data at follow-up (*n* = 3) or for missing values on alcohol consumption frequency (*n* = 8,841), CVD risk factors (*n* = 11,940), household data from the censuses (*n* = 25,656), and for education (*n* = 343). The final sample (*n* = 207,394) was included in complete case analyses using alcohol consumption frequency, of which 188,603 were current drinkers and 18,791 current abstainers. From this sample we also selected subgroups with data available on binge drinking episodes (*n* = 32,616) and data on lifetime abstaining (*n* = 30,455).

Individuals with missing values on household conditions were not different from those eligible and not different from individuals in the final sample. Individuals with missing alcohol data, and especially those with missing CVD risk factors, were older, more often female, had lower education, and experienced more CVD deaths during follow-up (S1 Table).

### Descriptive analyses

Baseline characteristics differed according to life course SEP for all included variables (Table 1). Participants with low SEP (*n* = 29,998) were on average older, more often female, had a higher prevalence of CVD risk factors, more previous diseases, and were more often a current abstainer or an infrequent consumer of alcohol. Participants with high SEP (*n* = 64,412) had the lowest prevalence of CVD risk factors, were more often frequent consumers of alcohol, and were more likely to binge drink within the subgroup for which these data were available. Estimates for middle SEP participants (*n* = 112,984) were mostly between the other strata.

**Table 1:** Descriptive characteristics of the study population at baseline according to categories of life course SEP.

	Life course SEP (*n* = 207,394)				
Variable	Low (*n* = 29,998)	Middle (*n* = 112,984)	High (*n* = 64,412)	*P-value*	*F* or χ^2^
Age	47.8 (11.7)	47.0 (10.8)	47.1 (11.0)	<0.001	73
Sex (male)	13,576 (45.3%)	54,313 (48.1%)	31,064 (52.1%)	<0.001	455
Education (1–8)	3.15 (1.36)	3.77 (1.55)	4.34 (1.58)	<0.001	6,481
Current smoker	12,547 (41.8%)	40,270 (35.6%)	19,466 (30.2%)	<0.001	1,282
Physical activity (1–4)	1.91 (0.90)	1.99 (0.91)	2.07 (0.91)	<0.001	322
BMI (kg/m^2^)	26.1 (4.23)	25.9 (3.91)	25.6 (3.68)	<0.001	205
Systolic blood pressure (mm Hg)	133.2 (19.5)	131.9 (18.3)	131.3 (17.9)	<0.001	113
Heart rate (bpm)	74.5 (12.5)	73.3 (12.4)	72.3 (12.3)	<0.001	338
Triglycerides (mmol/l)	1.80 (1.24)	1.74 (1.17)	1.70 (1.13)	<0.001	72
Total cholesterol (mmol/l)	5.83 (1.15)	5.79 (1.16)	5.75 (1.13)	<0.001	52
HDL-C (mmol/l)	1.35 (0.38)	1.37 (0.37)	1.38 (0.38)	<0.001	42
Females	1.45 (0.37)	1.49 (0.37)	1.52 (0.38)	<0.001	146
Males	1.23 (0.34)	1.24 (0.33)	1.25 (0.34)	<0.001	24
Diabetes	729 (2.4%)	1,989 (1.8%)	1,008 (1.6%)	<0.001	89
History of CVD	1,689 (5.6%)	5,054 (4.5%)	2,649 (4.1%)	<0.001	111
Family history of CHD	13,118 (43.7%)	48,302 (42.8%)	27,217 (42.1%)	0.001	18
Alcohol consumption frequency					
Current abstainer	3,197 (10.7%)	10,141 (9.0%)	5,423 (8.4%)	<0.001	126
Infrequent	8,643 (28.8%)	26,508 (23.5%)	12,212 (19.0%)	<0.001	1,182
Once per month to once per week	15,311 (51.0%)	62,041 (54.9%)	35,431 (55.0%)	<0.001	158
2–3 times per week	2,460 (8.2%)	12,546 (11.1%)	9,678 (15.0%)	<0.001	1,060
≥4 times per week	387 (1.3%)	1,748 (1.5%)	1,668 (2.6%)	<0.001	305
Average amount of alcohol (g/day)	3.9 (5.8)	4.3 (5.5)	4.9 (5.8)	<0.001	307
Heavy drinking episodes (*n* = 32,616)					
Not last year	2,205 (48.9%)	8,460 (48.3%)	4,992 (47.1%)	0.059	5.7
A few times	1,514 (33.6%)	5,971 (34.1%)	3,516 (33.2%)	0.256	2.7
1–3 times per month	628 (13.9%)	2,463 (14.1%)	1,638 (15.5%)	0.003	12
≥1 time per week	165 (3.7%)	609 (3.5%)	455 (4.3%)	0.002	12

Presented as mean (standard deviation) or count (percentages). Category means and frequencies were tested by analysis of variance and chi-squared test, respectively, and results are presented as *p*-values with affiliated statistic (*F*-value or chi-squared).

**Abbreviations:** BMI, body mass index; CHD, coronary heart disease; CVD, cardiovascular disease; HDL-C, high-density lipoprotein cholesterol; SEP, socioeconomic position

The distribution of covariates over categories of alcohol consumption frequency followed comparable patterns within the strata of life course SEP, but with different magnitudes (S4 Table). Notably, frequent consumers of alcohol were consistently more often also frequent bingers, but the percentage among the most frequent consumers who were also weekly bingers was 32.8% in the low, 19.1% in the middle, and 16.9% in the high SEP strata.

### Follow-up time and mortality

The mean (SD) follow-up time in the study population was 16.6 (4.0) years. In total, 25,950 participants died—8,435 (4.1%) from CVDs, including 3,837 from IHD and 1,972 from stroke. In the subgroup of current drinkers with additional data on heavy drinking episodes, 7,274 died during 15.4 (6.2) years of follow-up, 2,284 (7.0%) from CVDs, including 1,028 from IHD and 553 from stroke. In the subgroup with additional data on lifetime abstaining, the number of CVD deaths in the course of 12.5 (3.0) years was 2,166 (7.1%).

### Life course SEP and mortality

**Fig 2:**
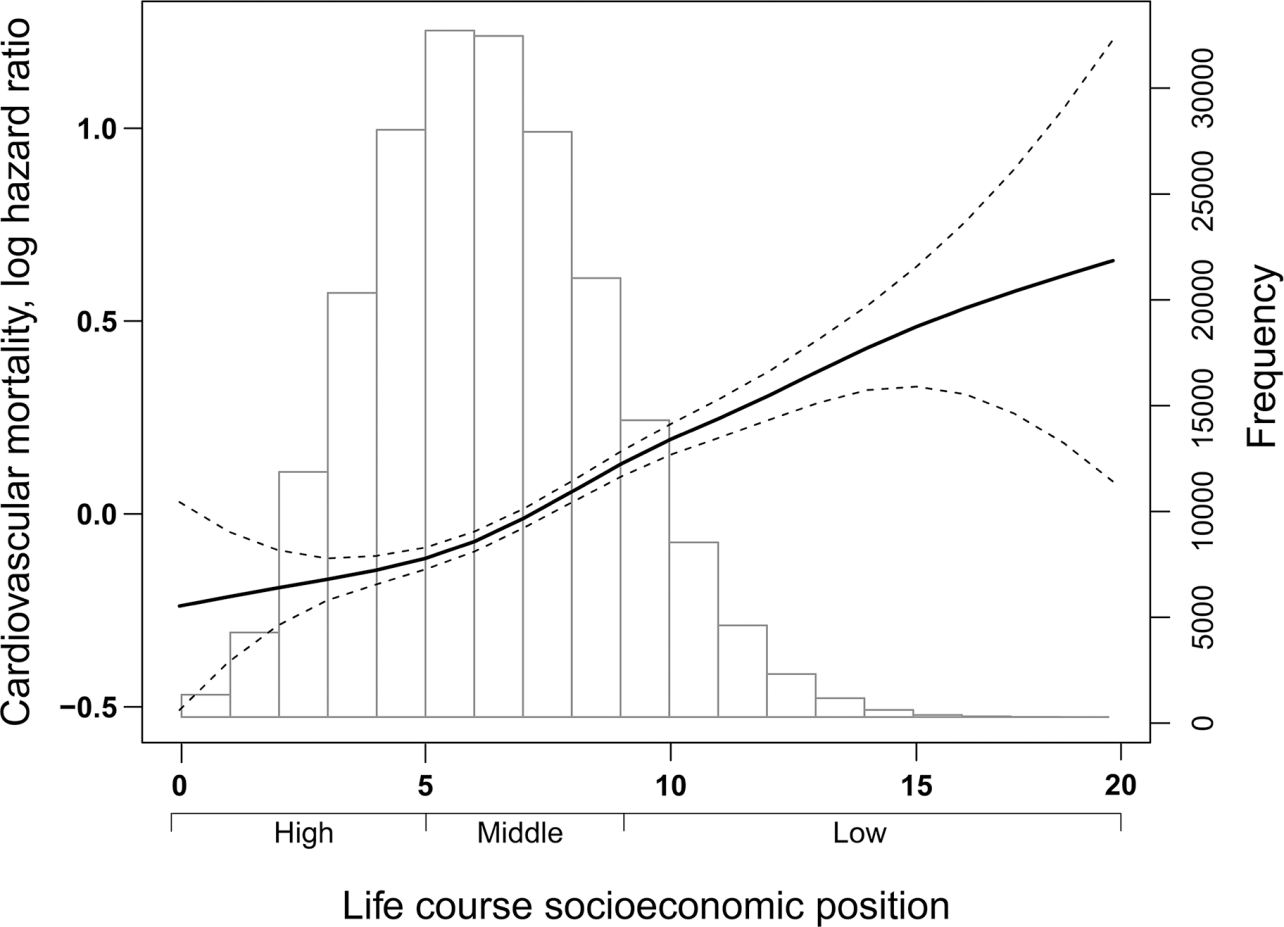
Frequency distribution of the study population (*n* = 207,394) according to the index of life course SEP (range 0–20) and (superimposed) the association of the index with the risk of cardiovascular mortality (8,435 deaths) during a mean (SD) follow-up of 16.6 (4.0) years. Cox proportional hazard model with life course socioeconomic index modelled as a penalised smoothing spline. The HR is on log scale and the relationship presented at the mean value of the covariates age and gender. HR, hazard ratio; SEP, socioeconomic position.

Fig 2 depicts the distribution of study participants according to the index of life course SEP and the dose-response relationship of the risk of CVD mortality with the index. The HR (and 95% CI) for risk of CVD mortality with each incremental increase of life course SEP index (range 0–20) was 1.06 (1.05, 1.07). In comparison to individuals with high SEP, HRs for risk of CVD mortality were 1.16 (1.11, 1.22) and 1.50 (1.41 1.59) among individuals with middle and low SEP, respectively.

### Alcohol consumption frequency, life course SEP, and mortality

The risk of CVD mortality was lower among frequent drinkers than among infrequent consumers in imputed (Table 2) and complete case analyses (S5 Table), with even lower estimates and stronger associations when excluding or adjusting for binge drinking (S6 Table). There was no difference in risk between infrequent consumers and lifetime abstainers when they were used as reference categories in a subgroup (S7 Table). Compared with infrequent drinkers, HRs among moderately frequent drinkers (2–3/week) were 0.78 (95% CI 0.72, 0.84) for CVD mortality (Table 2), 0.70 (95% CI 0.61, 0.79) for IHD mortality (S8 Table), 0.77 (95% CI 0.64, 0.93) for stroke mortality (S9 Table), and 0.91 (95% CI 0.87, 0.95) for all-cause mortality (S10 Table). Stratified analyses and tests for effect modification indicated differences in risk by life course SEP, in which HRs for CVD mortality, IHD mortality, and all-cause mortality were even lower among moderately frequent drinkers with high SEP than among moderately frequent drinkers with middle and low SEP. In the high, middle, and low strata of SEP respectively, HRs were 0.66 (95% CI 0.58, 0.76), 0.87 (95% CI 0.78, 0.97), and 0.79 (95% CI 0.64, 0.98) for CVD mortality, 0.53 (95% CI 0.42, 0.67), 0.83 (95% CI 0.70, 0.99), and 0.68 (95% CI 0.48, 0.97) for IHD mortality, and 0.86 (95% CI 0.79, 0.93), 0.94 (95% CI 0.88, 1.00), and 0.91 (95% CI 0.80, 1.03) for all-cause mortality. The analyses also indicated differences in risk by SEP for very frequent consumption (4–7/week). While HRs for very frequent consumption compared to infrequent consumption in the high and middle strata of SEP were 0.75 (95% CI 0.63, 0.90) and 0.77 (95% CI 0.64, 0.92) for CVD mortality, 1.08 (95% CI 0.76, 1.52) and 1.03 (95% CI 0.71, 1.48) for stroke mortality, and 0.93 (95% CI 0.84, 1.04) and 0.96 (95% CI 0.86, 1.06) for all-cause mortality, HRs for this level of drinking among participants with low SEP were 1.42 (95% CI 1.06, 1.90) for CVD mortality, 1.70 (95% CI 0.82, 3.51) for stroke mortality, and 1.49 (95% CI 1.24, 1.80) for all-cause mortality, respectively. HRs for IHD mortality in the high, middle, and low strata were 0.56 (95% CI 0.41, 0.77), 0.61 (95% CI 0.45, 0.84), and 0.87 (95% CI 0.50, 1.53), respectively.

**Table 2:** Cardiovascular mortality according to alcohol consumption frequency using multiple imputation (*n* = 245,336).

Life course SEP	Current drinkers (*n* = 220,726)				Current abstainer
*N* with/without event or pooled HRs (95% CIs) for CVD mortality	Infrequent (*n* = 58,217)	1/month to 1/week (*n* = 130,090)	2–3/week (*n* = 28,039)	4–7/week (*n* = 4,380)	(*n* = 24,610)
All	4,071/54,146	3,727/126,363	823/27,216	335/4,045	2,832/21,778
High	874/13,338	1,086/38,531	291/10,384	146/1,708	520/5,932
Middle	2,162/30,768	2,023/70,462	433/14,087	135/1,931	1,490/11,918
Low	1,035/10,040	618/17,370	99/2,745	54/406	822/3,928
Model 1					
All	1.00	0.82 (0.79, 0.86)	0.66 (0.61, 0.72)	0.71 (0.63, 0.79)	1.22 (1.16, 1.28)
High	1.00	0.79 (0.71, 0.86)	0.58 (0.50, 0.66)	0.62 (0.52, 0.74)	1.15 (1.02, 1.28)
Middle	1.00	0.88 (0.82, 0.94)	0.77 (0.70, 0.86)	0.75 (0.67, 0.90)	1.26 (1.17, 1.35)
Low	1.00	0.82 (0.74, 0.91)	0.75 (0.60, 0.92)	1.35 (1.01, 1.80)	1.18 (1.07, 1.30)
Model 2					
All	1.00	0.89 (0.85, 0.93)	0.78 (0.72, 0.84)	0.84 (0.75, 0.94)	1.22 (1.16, 1.28)
High	1.00	0.85 (0.77, 0.93)	0.66 (0.58, 0.76)	0.75 (0.63, 0.90)	1.16 (1.03, 1.30)
Middle	1.00	0.92 (0.86, 0.98)	0.87 (0.78, 0.97)	0.77 (0.64, 0.92)	1.28 (1.19, 1.37)
Low	1.00	0.84 (0.76, 0.94)	0.79 (0.64, 0.98)	1.42 (1.06, 1.90)	1.16 (1.05, 1.28)
Effect modification					
Middle versus high (ref)		1.08 (0.97, 1.21), *p* = 0.17	1.30 (1.10, 1.54), *p* = 0.002	1.01 (0.79, 1.30), *p* = 0.94	1.10 (0.96, 1.26), *p* = 0.16
Low versus high (ref)		1.03 (0.90, 1.18), *p* = 0.66	1.23 (0.96, 1.58), *p* = 0.10	1.90 (1.36, 2.66), *p* = 0.0001	1.00 (0.87, 1.16), *p* = 0.98
Low versus middle (ref)		0.95 (0.84, 1.08), *p* = 0.45	0.96 (0.76, 1.21), *p* = 0.73	1.81 (1.29, 2.54) *p* = 0.001	0.90 (0.80, 1.02), *p* = 0.09

*N* with or without event was the average from the 10 multiple imputed data sets and might not sum up exactly. Pooled HRs and 95% CIs were derived from Cox models. HRs among current drinkers (ordinal) and among current abstainers (dichotomous) were assessed in separate models, both with infrequent consumers as reference category. Models included (1) age and gender and (2) smoking, BMI, diabetes, physical activity, history of CVD, family history of CHD, systolic blood pressure, heart rate, triglycerides, and life course SEP (if not used as a stratifying variable). Effect modification (using model 2) was tested on a multiplicative scale and used the high or middle SEP stratum as a reference category.

**Abbreviations:** BMI, body mass index; CHD, coronary heart disease; CI, confidence interval; CVD, cardiovascular disease; HR, hazard ratio; *N*, number; ref, reference category; SEP, socioeconomic position

### Binge drinking, life course SEP, and mortality

Binge drinking (≥1 time/week) was associated with a higher risk of CVD mortality in imputed (Table 3) and complete case analysis (S11 Table) as well as a higher risk of IHD (S12 Table), and all-cause mortality (S14 Table) compared with no binge drinking the last year. HRs were 1.58 (95% CI 1.31, 1.91) for CVD mortality, 1.62 (95% CI 1.20, 2.17) for IHD mortality, 1.39 (95% CI 0.88, 2.20) for stroke mortality (S13 Table), and 1.36 (95% CI 1.20, 1.53) for all-cause mortality. HRs for less frequent binge drinking (1-3 times/month) were 1.12 (95% CI 0.98, 1.28) for CVD mortality, 1.09 (95% CI 0.88, 1.34) for IHD mortality, 1.15 (95% CI 0.86, 1.53) for stroke mortality, and 1.08 (95% CI 1.00, 1.17) for all-cause mortality, compared to no binge drinking the last year. Stratified analyses and tests for effect modification did not indicate large differences in risk by life course SEP. Estimates tended to be more robust and consistent in the larger middle stratum of SEP and less consistent in the low and high SEP strata.

**Table 3:** Cardiovascular mortality according to heavy drinking episodes using multiple imputation (*n* = 38,284).

Life course SEP		Current binge drinkers		
*N* with/without event or pooled HRs (95% CIs) for CVD mortality	Not last year (*n* = 18,438)	A few times last year (*n* = 12,900)	1–3 times per month (*n* = 5,499)	≥1 time per week (*n* = 1,447)
All	1,553/16,885	763/12,137	311/5,188	122/1,325
High	487/5,184	228/3,662	94/1,735	34/468
Middle	821/9,274	410/6,749	170/2,744	67/676
Low	245/2,427	125/1,726	46/709	22/182
Model 1				
All	1.00	1.06 (0.97, 1.16)	1.16 (1.02, 1.32)	1.79 (1.48, 2.16)
High	1.00	0.99 (0.84, 1.18)	0.94 (0.75, 1.19)	1.29 (0.90, 1.86)
Middle	1.00	1.11 (0.97, 1.26)	1.38 (1.16, 1.65)	2.22 (1.71, 2.88)
Low	1.00	1.07 (0.84, 1.37)	1.07 (0.76, 1.50)	1.95 (1.22, 3.10)
Model 2				
All	1.00	1.04 (0.95, 1.15)	1.12 (0.98, 1.28)	1.58 (1.31, 1.91)
High	1.00	0.97 (0.82, 1.16)	0.90 (0.71, 1.15)	1.22 (0.84, 1.76)
Middle	1.00	1.06 (0.93, 1.21)	1.31 (1.09, 1.57)	1.71 (1.31, 2.23)
Low	1.00	1.11 (0.87, 1.42)	0.99 (0.70, 1.41)	1.85 (1.16, 2.94)
Effect modification				
Middle versus high (ref)		1.04 (0.84, 1.27), *p* = 0.74	1.36 (1.02, 1.81), *p* = 0.04	1.36 (0.87, 2.13), *p* = 0.18
Low versus high (ref)		1.20 (0.91, 1.58), *p* = 0.20	1.18 (0.80, 1.73), *p* = 0.41	1.63 (0.92, 2.91), *p* = 0.10
Low versus middle (ref)		1.11 (0.86, 1.43), *p* = 0.42	0.89 (0.82, 1.29), *p* = 0.54	1.32 (0.79, 2.20), *p* = 0.29
Model 3				
All	1.00	1.11 (1.01, 1.22)	1.29 (1.12, 1.49)	1.92 (1.56, 2.36)
High	1.00	1.07 (0.90, 1.28)	1.12 (0.87, 1.44)	1.62 (1.09, 2.40)
Middle	1.00	1.11 (0.98, 1.27)	1.49 (1.22, 1.81)	2.04 (1.53, 2.71)
Low	1.00	1.16 (0.90, 1.50)	1.10 (0.76, 1.61)	1.82 (1.08, 3.08)

*N* with or without event was the average from the 10 multiple imputed data sets and might not sum up exactly. Pooled HRs and 95% CIs were derived from Cox models. Models included (1) age and gender, (2) smoking, BMI, diabetes, physical activity, history of CVD, family history of CHD, systolic blood pressure, heart rate, triglycerides, and life course SEP (if not used as a stratifying variable), and (3) the frequency of alcohol consumption. Effect modification (using model 2) was tested on a multiplicative scale and used the high or middle SEP stratum as reference category.

**Abbreviations:** BMI, body mass index; CHD, coronary heart disease; CI, confidence interval; CVD, cardiovascular disease; HR, hazard ratio; *N*, number; ref, reference category; SEP, socioeconomic position

### Additional analyses

The crude distributions of HDL-C according to categories of alcohol consumption frequency and life course SEP are presented (S4 Table). When adjusted for age and sex, the increase in HDL-C per increase in drinking frequency (4 frequency categories) was 0.093 (0.090, 0.095) mmol/l and corresponded to an estimated mean increase of approximately 26.6 g ethanol/day when we compared it to the estimated dose-response relationship between alcohol and HDL-C in a meta-analysis of experimental studies [17]. The increases in the high, middle, and low strata of SEP were 0.095 (0.090, 0.099), 0.090 (0.086, 0.093), and 0.086 (0.079, 0.093) mmol/l, respectively (*P-*interaction term = 0.715). In a subsample, the change in HDL-C per increase in the amount of alcohol consumed per day (grams/day) was 0.009 (0.009, 0.010) mmol/l. This corresponded to a 0.113 mmol/l increase per unit of alcohol, which is in the upper range when compared to experimental studies in which 1–2 drinks/day corresponded to an estimated increase in HDL-C of 0.072 mmol/l (0.024, 0.119) [17].

## Discussion

### Principal findings

Among adult participants in Norwegian health surveys (1987–2003), alcohol consumption and episodic heavy drinking were more frequent among individuals with high SEP throughout their life course. Participants with low SEP were more likely to currently abstain or drink less frequently, but apart from that, they were more exposed to all other CVD risk factors. Moderately frequent drinking was associated with a lower risk of CVD, IHD, and all-cause mortality than infrequent drinking, and we observed that this association was more pronounced among participants with high SEP. Very frequent drinking was associated with a higher risk of CVD and all-cause mortality compared with infrequent drinking, but only among participants with low SEP. Frequent binge drinking among current drinkers was associated with a higher risk of CVD, IHD, and all-cause mortality compared with no binge drinking during the last year, but it was not possible to determine whether the risk differed by life course SEP. Because of few events, it was also difficult to make firm inferences regarding stroke mortality.

### Interpretation of findings

The higher prevalence of current abstainers and infrequent consumers among those with low life course SEP is consistent with studies in other countries [35]. Alcohol taxes are particularly high in Norway, and differences in financial resources to purchase alcoholic beverages could contribute to this difference [36]. Interestingly, episodes of heavy drinking were somewhat more common among individuals with high SEP, illustrating the widespread acceptance of this behaviour in Norwegian society, even in the most health-conscious segment of the population. Another interesting observation was that individuals with low SEP were overrepresented in the most heavy drinking category (32.8%) when the frequencies of alcohol consumption and binge drinking were combined, a tendency that has also been observed previously in Europe [15]. The comparability to other populations in this regard strengthens external validity.

We observed lower risk of CVD, IHD, stroke, and all-cause mortality among moderately frequent drinkers in the study population compared with infrequent drinkers, which is in agreement with the majority of similar studies [16,37]. However, evidence from Mendelian randomisation studies thus far do not support a protective effect of alcohol on CVD nor provide support for a causal effect of factors that were considered to mediate a protective effect of alcohol—in particular, HDL-C and fibrinogen—on CVD [19,38–42]. It is therefore important to consider whether our findings may have been influenced by unmeasured confounders or misclassification of previous heavy drinkers [43]. In our study, we addressed the issue of reverse causality by choosing infrequent consumers over current abstainers as a reference category. We considered that small differences in alcohol consumption could not account for the difference in risk observed between these groups, unless moderate drinkers were misclassified as infrequent consumers because of underreporting [34,44]. Although we did not have data on lifetime abstainers for all participants, their risk of CVD did not differ from that of infrequent consumers in a subgroup analysis. We addressed the issue of confounding by adjusting for the uneven distribution of measured confounders, which did not strongly influence the associations. However, as measured confounders and thus, likely also unmeasured confounders, were distributed unevenly over categories of alcohol consumption within each stratum, there could clearly be residual confounding in the within-strata analyses as well. It is therefore unclear whether the findings reflect differential confounding of alcohol consumption with other exposures or differing effects of alcohol on health across socioeconomic groups.

The stratified analyses and tests for effect modification suggested that the relationship between alcohol consumption frequency and the risk of CVD mortality differed by life course SEP. The association between moderately frequent consumption and lower risk of CVD was more pronounced for participants with high SEP than among participants with low and middle SEP. Very frequent drinkers in the middle and high strata of SEP had either lower or comparable risk of CVD, IHD, stroke, and all-cause mortality in comparison to infrequent consumers, while very frequent drinkers with low SEP had a higher risk of CVD and all-cause mortality. Alcohol is subjected to first-pass metabolism in the gastrointestinal system, and when alcohol is co-ingested with foods, metabolism by enzymes in the stomach is extended [45]. This reduces bioavailability of ingested alcohol overall and also delays and reduces peak blood alcohol concentration, which may attenuate the systemic toxic effects of alcohol [46]. If drinking were more often accompanied by meals in one stratum of SEP, such as those with high SEP, it could account for a lower risk among binge drinkers compared to those not binge drinking, but not when considering alcohol as a protective factor. Another possibility, as indicated previously, is that the differences in risk by life course SEP could have arisen or been influenced by confounders having different effects in each stratum, such as if alcohol consumption is accompanied by a different set of behaviours in each stratum.

We observed higher CVD, IHD, and all-cause mortality among current drinkers who were frequent binge drinkers compared with current drinkers who did not binge drink during the last year, possibly mediated by increased blood pressure [19,47], cardiomyopathy, cardiac arrhythmias, and disturbances in blood electrolyte status [48,49]. A previous meta-analysis found binge drinking not to be associated with a higher risk of IHD in comparison to lifetime abstainers [50], and in that sense this finding sticks out. Findings were strong and consistent in the large middle stratum but less clear and less consistent in the smaller low and high SEP strata, which likely resulted in some inaccurate effect estimates and reduced precision when testing for effect modification. In light of the sample size and number of events, and the heterogeneity in the most extreme drinking categories, it is difficult to conclude with confidence that there is no socioeconomic difference in the relationship between binge drinking and CVD among adult Norwegians.

### Methodological considerations

Multiple measurements of alcohol exposure over time is the best approach to account for variation in consumption [51], but this study was limited to a single self-reported measurement. Previous studies found a test–retest correlation for men and women of approximately 0.6 when using data on repeated measurements from the source population [52,53]. Furthermore, 52% of the men and 62% of the women reported consistently in a follow-up postal survey 10 years later, of which abstainers were the most consistent (68% and 75%, respectively). Abstainers and heavy drinkers, however, appeared to be more prone to dropout than infrequent and light consumers. The large sample size also accounts for random variation.

We used HDL-C as a biomarker of a change in the magnitude of total alcohol consumption, and after adjusting for differences in age and sex, we observed an increase in HDL-C of about 0.093 mmol/l for each categorical increase in alcohol consumption frequency. This value corresponds to an estimated mean increase of approximately 26.6 g ethanol/day if we compare it to the estimated dose-response relationship between alcohol and HDL-C from a meta-analysis of clinical trials [17] and substantiated that increasing consumption frequency accompanied increased amount of ethanol consumed. The increase in amount of HDL-C was comparable within all strata of SEP; thus, it seems unlikely that differential exposure misclassification can explain the differing relationships between alcohol consumption and CVD mortality in the different strata of life course SEP. The dose-response relationship of cardiovascular mortality with alcohol consumption seemed to nadir at a frequency of 2–3 times per week, or 40 g ethanol/day higher intake on average than infrequent consumers, which is comparable to overall estimates from previous studies when men and women are combined [37]. The increase in HDL-C per increase in the amount of alcohol consumed was higher in the current study than in short-term experimental studies. Although the dose-response relationship between alcohol intake and HDL-C might differ for short-term and long-term intakes, the comparison suggests that alcohol consumption may be underreported to some extent in the health surveys.

Without information on previous alcohol intake or the cause of alcohol abstaining, we were unable to identify and exclude previous heavy drinkers. Only a few surveys distinguished between lifetime and current abstainers, which we combined with current abstainers in order to harmonise the data. As a result, findings involving abstainers have low generalisability. This also precluded the combination of infrequent drinkers and lifetime abstainers into a single comparison group, which has been suggested as the best alternative [51]. However, the sensitivity analysis comparing the use of lifetime abstainers and infrequent consumers did not indicate differences between these groups, which we consider a strength of our chosen reference category. Combined information on consumption frequency and volume were available for the subgroup with additional data on current episodes of heavy drinking. Apart from the overrepresentation of individuals with low SEP at the more extreme end of intake levels, there was a consistent increase in episodes of heavy drinking with increasing alcohol consumption frequency in all strata of life course SEP, suggesting that the main exposure variable, alcohol consumption frequency, differentiated individuals according to average alcohol intake.

To reflect life course SEP, we used an index constructed from multiple indicators that, with the exception of education, we derived from census surveys performed decennially between 1960 and 1990. In order for all participants to have the possibility to obtain a full score, we imposed selection criteria regarding immigration, birth date, time of death, and census participation. This resulted in a clearly defined and homogenous sample, but at the expense of sample size.

Previous studies have assessed the relationship between SEP and the risk of alcohol-related outcomes [54], and to various degrees, the mediating role of alcohol consumption [55]. Our study appears novel in the sense that it assesses the relationship between alcohol consumption and CVD mortality within strata of life course SEP, which appears to be very sparse or nonexistent in the current literature, regardless of how SEP is measured [55]. A possible reason could be the extensive sample size required to test for differences (effect modification) between groups and the registry linkages required to measure life course SEP, which highlights the major strengths of this study. For this reason and because of variation in alcohol consumption patterns, alcohol taxes, and socioeconomic inequalities between countries, it may be difficult to repeat the study in detail. The overall findings, however, should be available for replication in another population using similar study design, albeit with variation in the measurement of SEP or alcohol consumption.

### Conclusions

In this observational study of Norwegian adults, we observed lower CVD risk among frequent consumers of alcohol compared with infrequent consumers and higher CVD risk among current drinkers who reported frequent episodes of binge drinking in comparison to current drinkers who did not binge drink during the past year. The lower risk of CVD mortality associated with frequent consumption appeared to be more profound among those with high SEP throughout their life course than among those with middle and low SEP. We also observed higher CVD risk among very frequent consumers compared with infrequent consumers, but only among participants with low SEP. It was more uncertain whether the association between binge drinking and CVD mortality differed by life course SEP.

## Supporting information

S1 Checklist (DOCX)

S1 Protocol (PDF)

S1 Text Description of differences between the protocol and the study performed. (DOCX)

S1 Table The distribution of health survey participants and descriptive statistics in the source population, the population considered eligible, the study population, and groups excluded for missing values of either alcohol consumption frequency, cardiovascular risk factors, or indicators of life course socioeconomic position. (DOCX)

S2 Table Assessment of current abstaining and alcohol consumption frequency in the source surveys and harmonisation to construct the study variable. (DOCX)

S3 Table Assessment of the frequency of heavy drinking episodes in the source surveys and harmonisation to construct the study variable. (DOCX)

S4 Table Descriptive characteristics of the study population at baseline according to the frequency of alcohol consumption among participants with low (*n* = 29,998), middle (*n* = 112,984), and high (*n* = 64,412) life course socioeconomic position. (DOCX)

S5 Table Cardiovascular mortality according to alcohol consumption frequency in complete case analysis (*n* = 207,394). (DOCX)

S6 Table Cardiovascular mortality according to alcohol consumption frequency within the subgroup with data on heavy drinking episodes (*n* = 32,616). (DOCX)

S7 Table Cardiovascular mortality according to alcohol consumption frequency within the subgroup with data on lifetime abstaining (*n* = 30,455). (DOCX)

S8 Table Mortality from ischemic heart disease according to alcohol consumption frequency in the study population (*n* = 207,394) overall and in strata of life course socioeconomic position. (DOCX)

S9 Table Mortality from cerebrovascular disease (stroke) according to alcohol consumption frequency in the study population (*n* = 207,394) overall and in strata of life course socioeconomic position. (DOCX)

S10 Table All-cause mortality according to alcohol consumption frequency among current drinkers in the study population (*n* = 188,633). (DOCX)

S11 Table Cardiovascular mortality according to binge drinking episodes in complete case analysis (*n* = 33,534). (DOCX)

S12 Table Ischemic heart disease mortality according to binge drinking episodes in the subgroup with data on binge drinking (*n* = 32,616). (DOCX)

S13 Table Stroke mortality according to binge drinking episodes in the subgroup with data on binge drinking (*n* = 32,616). (DOCX)

S14 Table All-cause mortality according to binge drinking episodes in the subgroup (*n* = 32,616). (DOCX)

## Abbreviations

BMI: body mass index
CHD: coronary heart disease
CI: confidence interval
CVD: cardiovascular disease
HDL-C: high-density lipoprotein cholesterol
HR: hazard ratio
ICD: International Classification of Diseases
IHD: ischemic heart disease
N: number
PIN: personal identification number
ref: reference category
SEP: socioeconomic position
