# Supplementary material for: Life course socioeconomic position, alcohol drinking patterns in midlife, and cardiovascular mortality: Analysis of Norwegian population-based health surveys
Source: PLoS Med. 2018 Jan 2;15(1):e1002476. doi: 10.1371/journal.pmed.1002476 (PMC5749685; doi:10.1371/journal.pmed.1002476)
Supplement: S1 Checklist — (DOCX) [file pmed.1002476.s001.docx]

**The RECORD statement – checklist of items, extended from the STROBE statement, that should be reported in observational studies using routinely collected health data.**

|  | **Item No.** | **STROBE items** | **Location in manuscript where items are reported** | **RECORD items** | **Location in manuscript where items are reported** |
| --- | --- | --- | --- | --- | --- |
| **Title and abstract** | | | | | |
|  | 1 | (a) Indicate the study’s design with a commonly used term in the title or the abstract (b) Provide in the abstract an informative and balanced summary of what was done and what was found | (a) The study design is indicated in the method section, linking surveys (1987-2003) to follow-up in 2010.  (b) Methods and findings. | RECORD 1.1: The type of data used should be specified in the title or abstract. When possible, the name of the databases used should be included.  RECORD 1.2: If applicable, the geographic region and timeframe within which the study took place should be reported in the title or abstract.  RECORD 1.3: If linkage between databases was conducted for the study, this should be clearly stated in the title or abstract. | - 1. Name of data bases given in the method section of the manuscript, not the abstract, as there were many.   2. Norway and time period (1987-2003), including follow-up in number of years in average provided.   3. Included a sentence to indicate that the study uses data linkage. |
| **Introduction** | | | | | |
| Background rationale | 2 | Explain the scientific background and rationale for the investigation being reported | Ok, |  |  |
| Objectives | 3 | State specific objectives, including any prespecified hypotheses | Ok. We had no prespecified hypothesis to the direction of our results. |  |  |
| **Methods** | | | | | |
| Study Design | 4 | Present key elements of study design early in the paper | First paragraph indicates health surveys at one time point and follow-up through registry linkages. |  |  |
| Setting | 5 | Describe the setting, locations, and relevant dates, including periods of recruitment, exposure, follow-up, and data collection | Paragraph “*Study population*” |  |  |
| Participants | 6 | *(a) Cohort study* - Give the eligibility criteria, and the sources and methods of selection of participants. Describe methods of follow-up  *Case-control study* - Give the eligibility criteria, and the sources and methods of case ascertainment and control selection. Give the rationale for the choice of cases and controls  *Cross-sectional study* - Give the eligibility criteria, and the sources and methods of selection of participants  *(b) Cohort study* - For matched studies, give matching criteria and number of exposed and unexposed  *Case-control study* - For matched studies, give matching criteria and the number of controls per case | Paragraph “*Participants*” | RECORD 6.1: The methods of study population selection (such as codes or algorithms used to identify subjects) should be listed in detail. If this is not possible, an explanation should be provided.  RECORD 6.2: Any validation studies of the codes or algorithms used to select the population should be referenced. If validation was conducted for this study and not published elsewhere, detailed methods and results should be provided.  RECORD 6.3: If the study involved linkage of databases, consider use of a flow diagram or other graphical display to demonstrate the data linkage process, including the number of individuals with linked data at each stage. | The research protocol, and differences from this protocol for the study performed, is presented as text S1 and text S2, explaining in detail the data linkages and study populations. Also described in *Participants* and the flow chart *Figure 1*. |
| Variables | 7 | Clearly define all outcomes, exposures, predictors, potential confounders, and effect modifiers. Give diagnostic criteria, if applicable. | Paragraph “*Statistical analysis”* | RECORD 7.1: A complete list of codes and algorithms used to classify exposures, outcomes, confounders, and effect modifiers should be provided. If these cannot be reported, an explanation should be provided. | Extensive explanation for the exposure and outcome and other covariates. Also described in research protocol. |
| Data sources/ measurement | 8 | For each variable of interest, give sources of data and details of methods of assessment (measurement).  Describe comparability of assessment methods if there is more than one group | Provided for alcohol exposure, outcomes and for most covariates in *Covariates and outcome*. For life course socioeconomic position presented in the affiliated paragraph, with some additional information provided in the referenced study. |  |  |
| Bias | 9 | Describe any efforts to address potential sources of bias | We use HDL-cholesterol as a biomarker to test for underreporting and for differential misclassification in self-reported alcohol consumption (rational in *Statistical analysis* in the method section, results in *Additional analysis* in the result section page 12, and discussion under *Methodological considerations*). We also comment on reverse causality bias in the discussion and perform sensitivity analysis using different reference groups. |  |  |
| Study size | 10 | Explain how the study size was arrived at | A detailed flow chart is provided, figure 1. Also in paragraph *“Participants”.* |  |  |
| Quantitative variables | 11 | Explain how quantitative variables were handled in the analyses. If applicable, describe which groupings were chosen, and why | In the paragraphs *Alcohol exposure* and *Statistical analysis*. |  |  |
| Statistical methods | 12 | (a) Describe all statistical methods, including those used to control for confounding  (b) Describe any methods used to examine subgroups and interactions  (c) Explain how missing data were addressed  (d) *Cohort study* - If applicable, explain how loss to follow-up was addressed  *Case-control study* - If applicable, explain how matching of cases and controls was addressed  *Cross-sectional study* - If applicable, describe analytical methods taking account of sampling strategy  (e) Describe any sensitivity analyses | (a) In paragraph *“Statistical analysis.*  *(b)* Clearly stated how we test for effect modification. Subgroups described also in paragraph *Study population.*  (c) In statistical analysis. Listwise deletion and multiple imputation.  (e) described in the last part of the |  |  |
| Data access and cleaning methods |  |  |  | RECORD 12.1: Authors should describe the extent to which the investigators had access to the database population used to create the study population.  RECORD 12.2: Authors should provide information on the data cleaning methods used in the study. | 12.1 The research protocol, and differences from this protocol for the study performed, is presented as text S1 and text S2, explaining in detail the data linkages, how study populations were created from data bases, who performed the linkages, the third party. Also described in less detail in *Participants* and the flow chart *Figure 1*.  Presented in paragraph*“Data linkage”.*  12.2 Each registry performs quality control, the data is checked by a data manager, and the researcher clean data prior to analyses. |
| Linkage |  | .. |  | RECORD 12.3: State whether the study included person-level, institutional-level, or other data linkage across two or more databases. The methods of linkage and methods of linkage quality evaluation should be provided. | Presented in paragraph*“Data linkage” and “Participants”* informing that the study uses individual level data by the use of the personal identification number (PIN). |
| **Results** | | | | | |
| Participants | 13 | (a) Report the numbers of individuals at each stage of the study (*e.g.*, numbers potentially eligible, examined for eligibility, confirmed eligible, included in the study, completing follow-up, and analysed)  (b) Give reasons for non-participation at each stage.  (c) Consider use of a flow diagram | In *Participants* and in the flow chart (*figure 1*). | RECORD 13.1: Describe in detail the selection of the persons included in the study (*i.e.,* study population selection) including filtering based on data quality, data availability and linkage. The selection of included persons can be described in the text and/or by means of the study flow diagram. | In *Participants*, *data linkage,* and in the flow chart (*figure 1*), as well as in table S1. |
| Descriptive data | 14 | (a) Give characteristics of study participants (*e.g.*, demographic, clinical, social) and information on exposures and potential confounders  (b) Indicate the number of participants with missing data for each variable of interest  (c) *Cohort study* - summarise follow-up time (*e.g.*, average and total amount) | Both according to SEP (table 1) and according to alcohol within SEP strata (supplemental information, Table S4).  b) In *Participants* and in the flow chart (*figure 1*), and in table S1.  c) Paragraph “*Follow-up time and mortality”* |  |  |
| Outcome data | 15 | *Cohort study* - Report numbers of outcome events or summary measures over time  *Case-control study* - Report numbers in each exposure category, or summary measures of exposure  *Cross-sectional study* - Report numbers of outcome events or summary measures | Paragraph “*Follow-up time and mortality”* and tables 2 and 3, and S5 and S11 |  |  |
| Main results | 16 | (a) Give unadjusted estimates and, if applicable, confounder-adjusted estimates and their precision (e.g., 95% confidence interval). Make clear which confounders were adjusted for and why they were included  (b) Report category boundaries when continuous variables were categorized  (c) If relevant, consider translating estimates of relative risk into absolute risk for a meaningful time period | All estimates have 95% CI. Because of differences in age and sex, we defined this as the unadjusted estimates, because crude analyses would be misguiding. The model variables are provided in the method section, multiple paragraphs.  Ok.  We chose not to translate estimates of relative risk to absolute scale. |  |  |
| Other analyses | 17 | Report other analyses done—e.g., analyses of subgroups and interactions, and sensitivity analyses | As appropriate in the result section and in *Additional analysis* |  |  |
| **Discussion** | | | | | |
| Key results | 18 | Summarise key results with reference to study objectives | We present the principle findings initially. |  |  |
| Limitations | 19 | Discuss limitations of the study, taking into account sources of potential bias or imprecision. Discuss both direction and magnitude of any potential bias | We have tried to provide a balanced discussion of the most important limitations, primarily confined to *Methodological considerations,* but also in *Interpretation of findings* | RECORD 19.1: Discuss the implications of using data that were not created or collected to answer the specific research question(s). Include discussion of misclassification bias, unmeasured confounding, missing data, and changing eligibility over time, as they pertain to the study being reported. | We have assessed the possibility for reverse causality, underreporting, and non-differential and differential misclassification bias. It is explained as part of *Additional analysis* and discussed extensively throughout the discussion. |
| Interpretation | 20 | Give a cautious overall interpretation of results considering objectives, limitations, multiplicity of analyses, results from similar studies, and other relevant evidence | Ok. Spread throughout the discussion, depending on result. Mostly in paragraph *Interpretation of findings* |  |  |
| Generalisability | 21 | Discuss the generalisability (external validity) of the study results | An extensive discussion of different aspects of generalisability of the study results are given in multiple paragraphs in *Methodological considerations* |  |  |
| **Other Information** | | | | | |
| Funding | 22 | Give the source of funding and the role of the funders for the present study and, if applicable, for the original study on which the present article is based | Source of funding provided during submission. |  |  |
| Accessibility of protocol, raw data, and programming code |  | .. |  | RECORD 22.1: Authors should provide information on how to access any supplemental information such as the study protocol, raw data, or programming code. | We have given information on how to access raw data during submission. The research protocol, and any differences from this, is given in text S1 and S2. |

*Reference: Benchimol EI, Smeeth L, Guttmann A, Harron K, Moher D, Petersen I, Sørensen HT, von Elm E, Langan SM, the RECORD Working Committee. The REporting of studies Conducted using Observational Routinely-collected health Data (RECORD) Statement. *PLoS Medicine* 2015; in press.

*Checklist is protected under Creative Commons Attribution ([CC BY](http://creativecommons.org/licenses/by/4.0/)) license.
