# Supplementary material for: Life course socioeconomic position, alcohol drinking patterns in midlife, and cardiovascular mortality: Analysis of Norwegian population-based health surveys
Source: PLoS Med. 2018 Jan 2;15(1):e1002476. doi: 10.1371/journal.pmed.1002476 (PMC5749685; doi:10.1371/journal.pmed.1002476)
Supplement: S1 Text — (DOCX) [file pmed.1002476.s003.docx]

**S2 Text – Description of differences between the research protocol and the study performed.**

The study addresses one of several research questions in a larger project. The project has a protocol (in English) that describes the project aim, the specific research questions, central data sources, and the analysis plan. This document is included as (S1 Text). The project also has a technical document (only available in Norwegian) that describe all the data sources and the procedure for data linkage. These documents were part of the submission to the ethical committee, and subsequently in the application for funding to the Norwegian Research Council.

All decisions and details were not described in the protocol and some were made when starting to address this particular research question. It was also necessary to make some changes to the study population. Below we describe how the performed study adheres to the planned study in the project protocol:

- *Research question/aim:* The study addresses, in part, the following aim in the protocol: *“To investigate if the effects of moderate alcohol consumption and binge drinking on CVD can be explained by or interact with cognitive ability (IQ), educational level, or other socioeconomic factors”.* We have rephrased the research question in the manuscript, and use the word “differ” instead of “interact”, but the question is in essence the same. The question is clearly stated. IQ and education will be addressed in another manuscript.
- *Study population:* We did not obtain and include data from the twin registry in time to be included as planned. However, we were able to include data from the Age-40-program and the Counties studies. These data sources were not mentioned in the protocol, but are mentioned in the technical document (only available in Norwegian). The possibility to include and harmonise this data with the data in the Cohort of Norway was identified in the data cleaning process, it affects the project as a whole, and the decision to include the data was made before any analyses of this study. Accordingly, power calculations and some of the number presented in the protocol are no longer that relevant.
- S*tudy outcome*: Because of technical issues with accessing outcome data from hospital discharges on cardiovascular morbidity (FS-data), we decided to proceed with outcome data on cardiovascular mortality only (Cause of Death Registry). The CVD morbidity data will be available at a later stage in the project.
- *Alcohol consumption*: The variables from the Age 40 program and the Counties study are not describe in the protocol, but described in the manuscript.
- *Life course socioeconomic position*: The protocol states broadly which socioeconomic factors to include, and emphasises that a life course perspective should be used, but not how to include them in detail. We decided to use the index, which was created previously for another study in another project, and this decision was made before analysing the data (*Fiskå et al. Family history of premature myocardial infarction, life course socioeconomic position and coronary heart disease mortality - A Cohort of Norway (CONOR) study. International Journal of Cardiology 190 (2015) 302–307*).
- *Statistical analysis*:
  - The protocol stated that the relationship should be investigated using Cox models and to use interaction terms and stratification. These analytical methods are clearly described and we performed the analyses and report the findings accordingly.
  - Additional analyses in the manuscript included the evaluation of the socioeconomic index, which was deemed necessary and planned when we chose to use the index.
  - It is well established from RCTs that HDL increases upon alcohol consumption, and the first author was aware of this before conducting this study. However, the thought of using HDL to evaluate self-reporting overall and within each strata developed when performing univariate statistical analysis.. Hence, these analyses could be regarded as data driven. Therefore, we have revised the paragraph on “Statistical analysis”, and now begin the description of this analysis by stating: *“We also added an analysis after performing univariate descriptive statistical analysis”.* The decision to include the average amount of alcohol (g/day) was a result of the peer-review process

*Changes because of peer-review*: The revised manuscript state clearly that the inclusion of three secondary outcomes were the result of the peer-review
 (death from ischemic heart disease and stroke were requested). We decided to also include all-cause mortality. “*The Norwegian Cause of Death Registry provided outcome data on causes of death using ICD codes. The primary outcome was cardiovascular disease mortality using ICD codes (1990-1995: ICD-9 390-459; 1996-: ICD-10: I00-I99). Three secondary outcomes were added in response to peer-review, including death from ischemic heart disease (1990-1995: ICD-9 410-414; 1996-: ICD-10: I20-I25) and cerebrovascular diseases (1990-1995: ICD-9 430-438; 1996-: ICD-10: I60-I69), and all-cause mortality.”*
