# Supplementary material for: Life course socioeconomic position, alcohol drinking patterns in midlife, and cardiovascular mortality: Analysis of Norwegian population-based health surveys
Source: PLoS Med. 2018 Jan 2;15(1):e1002476. doi: 10.1371/journal.pmed.1002476 (PMC5749685; doi:10.1371/journal.pmed.1002476)
Supplement: S1 Table — (DOCX) [file pmed.1002476.s004.docx]

## **S1 Table.** The distribution of health survey participants and descriptive statistics in the source population, the population considered eligible, the study population, and groups excluded for missing values of either alcohol consumption frequency, cardiovascular risk factors, or indicators of life course socioeconomic position.

|  | |  | |  |  |  |  |  | **Excluded for missing values** | | | | |
| --- | --- | --- | --- | --- | --- | --- | --- | --- | --- | --- | --- | --- | --- |
| **Variable** | | **Potentially eligible  (n=317,171)** | |  | **Eligible (n=254,177)** |  | **Final sample  (n=207,394)** |  | **Alcohol**  **(n=8841)** |  | **CVD risk factors**  **(n=11,941)** |  | **Life course SEP**  **(n=25,656)** |
| Age | | 45.8 (12.4) | |  | 48.0 (11.8) |  | 47.1 (11.1) |  | 55.9 (14.8) |  | 60.9 (14.9) |  | 46.9 (11.2) |
| Sex (male), n (%) | | 152,329 (48.0) | |  | 131,073 (48.4) |  | 101,449 (48.9) |  | 3992 (45.2) |  | 5069 (42.4) |  | 12,421 (48.4) |
| Education (1-8) | | 3.90 (1.6) | |  | 3.77 (1.59) |  | 3.86 (1.59) |  | 3.01 (1.32) |  | 3.01 (1.34) |  | 3.68 (1.59) |
| CVD deaths, n (%) | | 14,024 (4.4) | |  | 13,149 (5.2) |  | 8435 (4.1) |  | 1361 (15.4) |  | 2045 (17.1) |  | 1291 (5.0) |
|  | |  | |  |  |  |  |  |  |  |  |  |  |
| **Health surveys** | | **N (%)*** | **Age (SD)** |  | **N (%)*** |  | **N (%)** |  | **N (%)** |  | **N (%)** |  | **N (%)** |
| ^a^ | Finnmark 3 | 17,860 (5.4) | 47.2 (9.3) |  | 15,994 (6.0) |  | 9512 (4.6) |  | 3165 (35.8) |  | 53 (0.4) |  | 1601 (6.2) |
|  | A40P94-97 | 73,723 (22.3) | 42.1 (5.3) |  | 66,621 (25.0) |  | 54,412 (26.2) |  | 243 (2.7) |  | 658 (5.5) |  | 6410 (25.0) |
|  | A40P97-99 | 66,361 (20.1) | 40.9 (1.2) |  | 59,534 (22.3) |  | 48,363 (23.3) |  | 732 (8.3) |  | 1210 (10.1) |  | 6794 (26.5) |
| ^a,b^ | Rest-Troms | 2479 (0.7) | 48.5 (13.8) |  | 1468 (0.6) |  | 1083 (0.5) |  | 29 (0.3) |  | 135 (1.1) |  | 212 (0.8) |
| ^a,b^ | Oslo 2 | 6873 (2.1) | 68.4 (6.2) |  | 6233 (2.3) |  | 5254 (2.5) |  | 88 (1.0) |  | 581 (4.9) |  | 304 (1.2) |
| ^a,b^ | HUBRO | 21,258 (6.4) | 48.0 (15.5) |  | 11,541 (4.3) |  | 9626 (4.6) |  | 119 (1.3) |  | 732 (6.1) |  | 1056 (4.1) |
| ^a,b,c^ | I-HUBRO | 3599 (1.1) | 38.5 (9.1) |  | - |  | - |  | - |  | - |  | - |
| ^a,b^ | OPPHED | 12,216 (3.7) | 49.6 (14.3) |  | 9153 (3.4) |  | 7638 (3.7) |  | 86 (1.0) |  | 423 (3.5) |  | 995 (3.9) |
|  | TROMSØ 4 | 19,751 (6.0) | 44.0 (15.0) |  | 13,236 (5.0) |  | 10,938 (5.3) |  | 51 (0.6) |  | 235 (2.0) |  | 1959 (7.6) |
|  | \|HUSK | 25,462 (7.7) | 47.1 (9.8) |  | 23,195 (8.9) |  | 19,762 (9.5) |  | 600 (6.8) |  | 821 (6.9) |  | 1982 (7.7) |
| ^a,b^ | TROMSØ 5 | 7832 (2.4) | 59.3 (14.1) |  | 6215 (2.3) |  | 4601 (2.2) |  | 229 (2.6) |  | 677 (5.7) |  | 687 (2.7) |
| ^a,b^ | REST-FINN | 6492 (2.0) | 58.9 (11.0) |  | 5532 (2.1) |  | 1352 (0.7) |  | 15 (0.2) |  | 194 (1.6) |  | 237 (0.9) |
| ^d^ | HUNT 2 | 64,889 (19.6) | 49.9 (17.4) |  | 46,884 (17.6) |  | 33,952 (16.4) |  | 3460 (39.1) |  | 6103 (51.1) |  | 3338 (13.0) |
| ^a,b^ | MORO 2 | 1905 (0.6) | 51.1 (9.6) |  | 1136 (0.4) |  | 901 (0.4) |  | 24 (0.3) |  | 128 (1.1) |  | 81 (0.3) |

Abbreviations: CVD=cardiovascular disease; SEP=socioeconomic position.
* Number and percentages for the distribution of health surveys are based on participants observations (n=330,300 and n=266,742, respectively).

^a)^ Assessed binge drinking episodes. ^b)^ Assessed life time abstaining. ^c)^ I-HUBRO included immigrants only and was not eligible for the study. ^d)^ Physical activity accounted for the high number of missing in HUNT 2.
