# Supplementary material for: Life course socioeconomic position, alcohol drinking patterns in midlife, and cardiovascular mortality: Analysis of Norwegian population-based health surveys
Source: PLoS Med. 2018 Jan 2;15(1):e1002476. doi: 10.1371/journal.pmed.1002476 (PMC5749685; doi:10.1371/journal.pmed.1002476)
Supplement: S2 Table — (DOCX) [file pmed.1002476.s005.docx]

## **S2 Table.** Assessment of current abstaining and alcohol consumption frequency in the source surveys and harmonisation to construct the study variable.

|  |  | **Alcohol consumption frequency** | | | | |
| --- | --- | --- | --- | --- | --- | --- |
|  |  | **The Counties study – Finnmark III^1^** |  | **Age 40 Program**  **CONOR^2^** |  | **CONOR^2^** |
| *Study variable* |  | *How often do you consume alcohol?* |  | *How many times per month do you consume alcohol?* |  | *How often during the past 12 months have you consumed alcohol?* |
|  |  |  |  |  |  |  |
| 4-7 times per week |  | Daily |  | ≥16 |  | 4-7 times per week |
| 2-3 times per week |  | 2-3 times per week |  | *7-15* |  | 2-3 times per week |
| Once/month – once/week |  | Once per week |  | 4-6 |  | Once per week |
|  |  | 1-2 times a month |  | 1-3 |  | 1-3 times per month |
| Infrequent |  | A few times per year |  | 0 |  | A few times/not last year |
| Current abstainer |  | Current abstainer |  | Current abstainer |  | Lifetime abstainer |
|  |  |  |  |  |  |  |

^1^ Consumption frequencies of beer, wine, and liquor were assessed in separate questions and summed to reflect total frequency.

^2^ Assessment differed between surveys within CONOR, of which 3 corresponded to assessment in the Age 40 Program.
