# Supplementary material for: Life course socioeconomic position, alcohol drinking patterns in midlife, and cardiovascular mortality: Analysis of Norwegian population-based health surveys
Source: PLoS Med. 2018 Jan 2;15(1):e1002476. doi: 10.1371/journal.pmed.1002476 (PMC5749685; doi:10.1371/journal.pmed.1002476)
Supplement: S3 Table — (DOCX) [file pmed.1002476.s006.docx]

## **S3 Table.** Assessment of the frequency of heavy drinking episodes in the source surveys and harmonisation to construct the study variable.

|  | **Frequency of heavy drinking episodes** | | |
| --- | --- | --- | --- |
|  | **The Counties study**  **– Finnmark III** |  | **CONOR** |
| *Study variable* | How often during the past year did you drink at least the equivalent of 5 small bottles of beer, a full bottle of wine or a ¼ bottle of liquor? |  | Approximately how often during the past 12 months have you consumed alcohol corresponding to at least 5 glasses and/or spirits in 24 hours? |
|  |  |  |  |
| ≥1 time per week | ≥1 time per week |  | 37-99 |
| 1-3 times per month | 1-3 times per month |  | 10-36 |
| A few times | A few times |  | 1-9 |
| Not last year | Not last year |  | 0 |
|  |  |  |  |
