# Supplementary material for: Life course socioeconomic position, alcohol drinking patterns in midlife, and cardiovascular mortality: Analysis of Norwegian population-based health surveys
Source: PLoS Med. 2018 Jan 2;15(1):e1002476. doi: 10.1371/journal.pmed.1002476 (PMC5749685; doi:10.1371/journal.pmed.1002476)
Supplement: S4 Table — (DOCX) [file pmed.1002476.s007.docx]

## **S4 Table.** Descriptive characteristics of the study population at baseline according to the frequency of alcohol consumption among participants with low (n=29,998), middle (n=112,984), and high (n=64,412) life course socioeconomic position.

|  | |  |  | **Alcohol consumption frequency** | | | |
| --- | --- | --- | --- | --- | --- | --- | --- |
| **Variables** | | **Strata of life course SEP** | **Current**  **abstainer** | **Infrequent** | **Once per month to once per week** | **2-3 times**  **per week** | **≥4 times**  **per week** |
| Participants, % | | L | 3197 (10.7 %) | 8643 (28.8 %) | 15,311 (28.8 %) | 2460 (8.2 %) | 383 (1.2 %) |
|  | | M | 10,141 (9.0 %) | 26,508 (23.5 %) | 62,041 (54.9 %) | 12,546 (11.1 %) | 1748 (1.5 %) |
|  | | H | 5423 (8.4 %) | 12,212 (19.0 %) | 35,431 (55.0 %) | 9678 (15.0 %) | 1668 (2.6%) |
| Age | | L | 54.8 (15.3) | 50.3 (13.3) | 45.2 (9.0) | 46.2 (9.5) | 52.6 (12.2) |
|  | | M | 51.3 (13.9) | 49.2 (12.5) | 45.2 (8.9) | 46.4 (9.6) | 53.7 (13.0) |
|  | | H | 49.5 (13.1) | 49.0 (12.5) | 45.5 (9.6) | 47.2 (10.6) | 57.1 (13.7) |
| Sex (male) | | L | 899 (28.1 %) | 3144 (36.4 %) | 7677 (50.1 %) | 1561 (63.5 %) | 295 (76.2 %) |
|  | | M | 3393 (33.5 %) | 9519 (35.9 %) | 32,126 (51.8 %) | 8021 (63.9 %) | 1254 (71.7 %) |
|  | | H | 2203 (40.6 %) | 4685 (38.4 %) | 19,129 (54.0 %) | 6314 (65.2 %) | 1229 (73.7 %) |
| Education (1-8) | | L | 2.83 (1.30) | 2.89 (1.22) | 3.27 (1.43) | 3.69 (1.64) | 3.65 (1.75) |
|  | | M | 3.44 (1.55) | 3.39 (1.41) | 3.85 (1.53) | 4.35 (1.67) | 4.45 (1.73) |
|  | | H | 4.04 (1.59) | 3.91 (1.48) | 4.35 (1.54) | 4.89 (1.60) | 5.06 (1.63) |
| Current smoker | | L | 680 (21.3 %) | 3489 (40.4 %) | 7101 (46.4 %) | 1085 (44.1 %) | 192 (49.6 %) |
|  | | M | 1728 (17.0 %) | 9193 (34.7 %) | 24,088 (38.8 %) | 4546 (36.2 %) | 715 (40.9 %) |
|  | | H | 653 (12.0 %) | 3453 (28.3 %) | 11,762 (33.2 %) | 3097 (32.0 %) | 501 (30.0 %) |
| Physical activity | | L | 1.67 (0.87) | 1.79 (0.87) | 2.00 (0.90) | 2.09 (0.88) | 1.90 (0.82) |
|  | | M | 1.78 (0.87) | 1.86 (0.89) | 2.05 (0.91) | 2.15 (0.90) | 2.02 (0.86) |
|  | | H | 1.88 (0.89) | 1.92 (0.90) | 2.11 (0.91) | 2.20 (0.90) | 2.09 (0.89) |
| Body mass index (kg/m^2^) | | L | 26.91 (4.93) | 26.38 (4.59) | 25.93 (3.91) | 25.62 (3.63) | 25.88 (3.93) |
|  | | M | 26.50 (4.50) | 26.19 (4.31) | 25.71 (3.71) | 25.51 (3.41) | 25.58 (3.58) |
|  | | H | 26.11 (4.21) | 25.91 (4.12) | 25.52 (3.54) | 25.32 (3.28) | 25.45 (3.29) |
| Systolic blood pressure (mm Hg) | | L | 141 (23.8) | 135 (20.9) | 131 (17.4) | 132 (17.7) | 139 (20.0) |
|  | | M | 136 (22.0) | 134 (19.8) | 131 (16.9) | 132 (16.8) | 138 (19.3) |
|  | | H | 133 (20.3) | 133 (19.4) | 130 (16.9) | 132 (17.0) | 138 (20.0) |
| Heart rate (bpm) | | L | 75.8 (13.4) | 75.6 (12.8) | 73.9 (12.1) | 72.4 (12.2) | 76.0 (13.5) |
|  | | M | 74.7 (13.1) | 74.7 (12.6) | 72.8 (12.1) | 71.7 (12.3) | 73.2 (13.0) |
|  | | H | 73.4 (12.7) | 73.8 (12.5) | 72.0 (12.1) | 71.1 (12.2) | 71.4 (12.4) |
| Triglycerides (mmol/l) | | L | 1.84 (1.28) | 1.80 (1.26) | 1.78 (1.18) | 1.82 (1.43) | 1.92 (1.47) |
|  | | M | 1.79 (1.15) | 1.74 (1.16) | 1.74 (1.19) | 1.73 (1.16) | 1.74 (1.27) |
|  | | H | 1.74 (1.10) | 1.69 (1.06) | 1.70 (1.13) | 1.70 (1.24) | 1.67 (1.03) |
| Total cholesterol (mmol/l) | | L | 6.08 (1.29) | 5.89 (1.20) | 5.75 (1.09) | 5.80 (1.09) | 5.97 (1.17) |
|  | | M | 5.98 (1.57) | 5.86 (1.19) | 5.72 (1.09) | 5.78 (1.06) | 5.95 (1.10) |
|  | | H | 5.85 (1.21) | 5.82 (1.19) | 5.70 (1.08) | 5.77 (1.15) | 6.01 (1.12) |
| HDL-cholesterol (mmol/l) | | L | 1.35 (0.38) | 1.33 (0.36) | 1.35 (0.37) | 1.42 (0.40) | 1.49 (0.47) |
|  | | M | 1.34 (0.37) | 1.34 (0.36) | 1.37 (0.37) | 1.43 (0.39) | 1.53 (0.43) |
|  | | H | 1.32 (0.36) | 1.35 (0.37) | 1.37 (0.37) | 1.45 (0.41) | 1.59 (0.47) |
| Diabetes | | L | 169 (5.3 %) | 272 (3.1 %) | 241 (1.6 %) | 31 (1.3 %) | 16 (4.1 %) |
|  | | M | 383 (3.8 %) | 693 (2.6 %) | 754 (1.2 %) | 120 (1.0 %) | 39 (2.2 %) |
|  | | H | 143 (2.6 %) | 293 (2.4 %) | 410 (1.2 %) | 117 (1.2 %) | 45 (2.7 %) |
| History of CHD or stroke | | L | 355 (11.1 %) | 639 (7.4 %) | 567 (3.7 %) | 97 (3.9 %) | 31 (8.0 %) |
|  | | M | 782 (7.7 %) | 1655 (6.2 %) | 2022 (3.3 %) | 440 (3.5 %) | 152 (8.7 %) |
|  | | H | 367 (6.8 %) | 707 (5.8 %) | 1080 (3.0 %) | 337 (3.5 %) | 158 (9.5 %) |
| Family history of CHD | | L | 1409 (44.1 %) | 3808 (44.1 %) | 6742 (44.0 %) | 1007 (40.9 %) | 152 (39.3 %) |
|  | | M | 4421 (43.6 %) | 11,653 (44.0 %) | 26,420 (42.6 %) | 5064 (40.4 %) | 744 (42.6 %) |
|  | | H | 2418 (44.6 %) | 5416 (44.3 %) | 14,801 (41.8 %) | 3958 (40.9 %) | 624 (37.4 %) |
| Average amount of alcohol (g/day) | | L | - | 0.24 (0.90) | 4.25 (3.86) | 12.0 (7.46) | 22.5 (16.5) |
|  | | M | - | 0.23 (0.99) | 4.20 (3.57) | 11.4 (6.61) | 18.6 (12.3) |
|  | | H | - | 0.23 (0.94) | 4.25 (3.51) | 11.3 (6.27) | 17.3 (10.5) |
| Heavy drinking episodes | |  |  |  |  |  |  |
|  | Not last year | L | - | 948 (75.4 %) | 1048 (43.1 %) | 167 (26.1 %) | 42 (23.0 %) |
|  |  | M | - | 2981 (74.9 %) | 4294 (45.6 %) | 916 (28.4 %) | 269 (30.4 %) |
|  |  | H | - | 1434 (77.6 %) | 2519 (47.0 %) | 772 (31.0 %) | 267 (29.8 %) |
|  | A few times | L | - | 309 (24.6 %) | 956 (39.3 %) | 212 (33.1 %) | 37 (20.2 %) |
|  |  | M | - | 980 (24.6 %) | 3722 (39.5 %) | 1060 (32.8 %) | 209 (23.6 %) |
|  |  | H | - | 408 (22.1 %) | 2078 (38.7 %) | 805 (32.3 %) | 225 (25.1 %) |
|  | 1-3 times per month | L | - | 1 (0.1 %) | 389 (16.0 %) | 194 (30.3 %) | 44 (24.0 %) |
|  |  | M | - | 15 (0.4 %) | 1262 (13.4 %) | 947 (29.3 %) | 239 (27.0 %) |
|  |  | H | - | 7 (0.4 %) | 685 (12.8 %) | 694 (27.8 %) | 252 (28.2 %) |
|  | ≥1 time per week | L | - | 0 (0.0 %) | 38 (1.6 %) | 67 (10.5 %) | 60 (32.8 %) |
|  |  | M | - | 2 (0.1 %) | 133 (1.4 %) | 305 (9.4 %) | 169 (19.1 %) |
|  |  | H | - | 0 (0.0 %) | 83 (1.5 %) | 221 (8.9 %) | 151 (16.9 %) |

Abbreviations: L=Low; M=Middle; H=High; CHD=coronary heart disease. Provided as mean (standard deviation) or counts (percentages). Data on the frequency of heavy drinking episodes was available for 4512, 17,503 and 10,601 subjects in the strata (L, M, H).
