# Supplementary material for: Life course socioeconomic position, alcohol drinking patterns in midlife, and cardiovascular mortality: Analysis of Norwegian population-based health surveys
Source: PLoS Med. 2018 Jan 2;15(1):e1002476. doi: 10.1371/journal.pmed.1002476 (PMC5749685; doi:10.1371/journal.pmed.1002476)
Supplement: S7 Table — (DOCX) [file pmed.1002476.s010.docx]

## **S7 Table.** Cardiovascular mortality according to alcohol consumption frequency within the subgroup with data on lifetime abstaining (n=30,455).

| **Life course SEP** | |  |  |  | **Current drinkers (n=30,455)** | | | |
| --- | --- | --- | --- | --- | --- | --- | --- | --- |
| N with/without event or  HR (95% CI) for CVD mortality | |  | **Lifetime  abstainer (n=1149)** |  | **Infrequent (n=7466)** | **1/month to 1/week  (n=14,611)** | **2-3/week (n=5352)** | **4-7/week (n=1877)** |
|  | All |  | 128/1021 |  | 766/6700 | 812/13,799 | 301/5051 | 159/1718 |
|  | High |  | 33/291 |  | 173/1509 | 250/3985 | 99/1943 | 70/795 |
|  | Middle |  | 66/552 |  | 419/3729 | 448/7655 | 162/2544 | 64/761 |
|  | Low |  | 29/178 |  | 174/1462 | 114/2159 | 40/564 | 25/162 |
| Model 1 | |  |  |  |  |  |  |  |
|  | All |  | 1.00 |  | 1.10 (0.91, 1.33) | 0.82 (0.67, 0.99) | 0.71 (0.57, 0.87) | 0.74 (0.59, 0.94) |
|  | High |  | 1.00 |  | 1.23 (0.85, 1.78) | 0.93 (0.65, 1.35) | 0.65 (0.43, 0.96) | 0.73 (0.48, 1.11) |
|  | Middle |  | 1.00 |  | 1.09 (0.84, 1.41) | 0.83 (0.64, 1.08) | 0.82 (0.61, 1.10) | 0.76 (0.54, 1.08) |
|  | Low |  | 1.00 |  | 0.93 (0.62, 1.38) | 0.67 (0.44, 1.02) | 0.75 (0.46, 1.22) | 1.33 (0.77, 2.31) |
| Model 2 | |  |  |  |  |  |  |  |
|  | All |  | 1.00 |  | 1.02 (0.84, 1.23) | 0.83 (0.69, 1.01) | 0.75 (0.61, 0.93) | 0.81 (0.63, 1.02) |
|  | High |  | 1.00 |  | 1.10 (0.76, 1.60) | 0.89 (0.61, 1.28) | 0.62 (0.42, 0.93) | 0.69 (0.45, 1.05) |
|  | Middle |  | 1.00 |  | 1.06 (0.73, 1.54) | 0.88 (0.61, 1.27) | 0.62 (0.41, 0.92) | 0.72 (0.47, 1.10) |
|  | Low |  | 1.00 |  | 1.00 (0.77, 1.30) | 0.82 (0.63, 1.07) | 0.81 (0.61, 1.10) | 0.72 (0.50, 1.02) |
| Model 2* | |  |  |  |  |  |  |  |
|  | All |  | 0.98 (0.81, 1.18) |  | 1.00 | 0.82 (0.74, 0.91) | 0.74 (0.64, 0.85) | 0.79 (0.66, 0.94) |
|  | High |  | 0.94 (0.65, 1.38) |  | 1.00 | 0.83 (0.68, 1.01) | 0.58 (0.45, 0.75) | 0.68 (0.51, 0.90) |
|  | Middle |  | 1.00 (0.77, 1.30) |  | 1.00 | 0.82 (0.72, 0.94) | 0.82 (0.68, 0.98) | 0.72 (0.55, 0.94) |
|  | Low |  | 1.05 (0.70, 1.58) |  | 1.00 | 0.73 (0.57, 0.93) | 0.87 (0.61, 1.24) | 1.53 (0.99, 2.35) |

Abbreviations: CVD=cardiovascular disease; SEP=socioeconomic position. Hazard ratios (HRs) and 95% confidence intervals (CIs) derived from Cox models with alcohol as an ordinal variable and lifetime abstainer as reference category. Models (1) included age and gender, and (2) smoking, body mass index, diabetes, physical activity, history of CVD, family history of coronary heart disease, systolic blood pressure, heart rate, triglycerides, and life course SEP (if not used as a stratifying variable. * Changed reference category.
