# Supplementary material for: Life course socioeconomic position, alcohol drinking patterns in midlife, and cardiovascular mortality: Analysis of Norwegian population-based health surveys
Source: PLoS Med. 2018 Jan 2;15(1):e1002476. doi: 10.1371/journal.pmed.1002476 (PMC5749685; doi:10.1371/journal.pmed.1002476)
Supplement: S9 Table — (DOCX) [file pmed.1002476.s012.docx]

## **S9 Table.** Mortality from cerebrovascular disease (stroke) according to alcohol consumption frequency in the study population (n=207,394) overall and in strata of life course socioeconomic position.

| **Life course SEP** | |  | **Current drinkers** | | | | |  | **Current abstainer** |
| --- | --- | --- | --- | --- | --- | --- | --- | --- | --- |
| N with/without event or  HR (95% CI) for stroke mortality | |  | **Infrequent (n=47,363)** |  | **1/month to 1/week  (n=112,783)** | **2-3/week (n=24,684)** | **4-7/week (n=3803)** |  | **(n=18,761)** |
|  | All |  | 645/46,718 |  | 684/112,099 | 147/24,537 | 83/3720 |  | 413/18,348 |
|  | High |  | 166/12,046 |  | 231/35,200 | 62/9616 | 43/1625 |  | 101/5322 |
|  | Middle |  | 350/23,158 |  | 356/61,685 | 72/12,474 | 32/1716 |  | 206/9935 |
|  | Low |  | 129/8514 |  | 97/15,214 | 13/2447 | 8/379 |  | 106/3091 |
| Model 1 | |  |  |  |  |  |  |  |  |
|  | All |  | 1.00 |  | 0.91 (0.81, 1.01) | 0.70 (0.58, 0.84) | 1.02 (0.81, 1.29) |  | 1.19 (1.05, 1.34) |
|  | High |  | 1.00 |  | 0.88 (0.71, 1.08) | 0.64 (0.47, 0.86) | 0.95 (0.67, 1.34) |  | 1.17 (0.91, 1.49) |
|  | Middle |  | 1.00 |  | 0.92 (0.79, 1.07) | 0.77 (0.60, 1.00) | 1.03 (0.71, 1.48) |  | 1.14 (0.95, 1.35) |
|  | Low |  | 1.00 |  | 0.96 (0.73, 1.27) | 0.70 (0.39, 1.25) | 1.50 (0.73, 3.09) |  | 1.31 (1.00, 1.71) |
| Model 2 | |  |  |  |  |  |  |  |  |
|  | All |  | 1.00 |  | 0.95 (0.85, 1.07) | 0.77 (0.64, 0.93) | 1.12 (0.89, 1.42) |  | 1.19 (1.04, 1.35) |
|  | High |  | 1.00 |  | 0.92 (0.75, 1.14) | 0.70 (0.52, 0.95) | 1.08 (0.76, 1.52) |  | 1.17 (0.91, 1.51) |
|  | Middle |  | 1.00 |  | 0.95 (0.82, 1.11) | 0.84 (0.64, 1.09) | 1.03 (0.71, 1.48) |  | 1.15 (0.96, 1.37) |
|  | Low |  | 1.00 |  | 1.00 (0.75, 1.32) | 0.76 (0.42, 1.36) | 1.70 (0.82, 3.51) |  | 1.29 (0.98, 1.68) |
| Effect modification | |  |  |  |  |  |  |  |  |
|  | Middle vs high (ref) |  |  |  | 1.05 (0.82, 1.34), p=0.72 | 1.19 (0.81, 1.75), p=0.37 | 0.95 (0.58, 1.55), p=0.83 |  | 0.97 (0.72, 1.32), p=0.87 |
|  | Low vs high (ref) |  |  |  | 1.12 (0.80, 1.56), p=0.51 | 1.06 (0.56, 2.01), p=0.86 | 1.49 (0.68, 3.29), p=0.32 |  | 1.11 (0.77, 1.58), p=0.58 |
|  | Low vs middle (ref) |  |  |  | 1.07 (0.79, 1.44), p=0.67 | 0.89 (0.48, 1.66), p=0.72 | 1.57 (0.71, 3.51), p=0.27 |  | 1.14 (0.83, 1.55), p=0.42 |

Abbreviations: SEP=socioeconomic position. Hazard ratios (HRs) and 95% confidence intervals (CIs) derived from Cox models. HRs among current drinkers (ordinal) and among current abstaining (dichotomous) were assessed in separate models, both with infrequent consumers as reference category. Models (1) included age and gender, and (2) smoking, body mass index, diabetes, physical activity, history of CVD, family history of coronary heart disease, systolic blood pressure, heart rate, triglycerides, and life course SEP (if not used as a stratifying variable). Effect modification (using model 2) was tested on a multiplicative scale and used the high or middle SEP strata as reference category.
