# Supplementary material for: Life course socioeconomic position, alcohol drinking patterns in midlife, and cardiovascular mortality: Analysis of Norwegian population-based health surveys
Source: PLoS Med. 2018 Jan 2;15(1):e1002476. doi: 10.1371/journal.pmed.1002476 (PMC5749685; doi:10.1371/journal.pmed.1002476)
Supplement: S10 Table — (DOCX) [file pmed.1002476.s013.docx]

## **S10 Table.** All-cause mortality according to alcohol consumption frequency among current drinkers (n=188,633).

| **Life course SEP** | |  | **Current drinkers** | | | | |
| --- | --- | --- | --- | --- | --- | --- | --- |
| N with/without event or HR (95% CI) for all-cause mortality | |  | **Infrequent (n=47,363)** |  | **1/month to 1/week  (n=112,783)** | **2-3/week (n=24,684)** | **4-7/week (n=3803)** |
|  | All |  | 8019/39,344 |  | 10,124/102,659 | 2536/22,148 | 945/2858 |
|  | High |  | 1932/10,280 |  | 3151/32,280 | 1023/8655 | 440/1228 |
|  | Middle |  | 4318/22,190 |  | 5391/56,650 | 1230/11,316 | 384/1364 |
|  | Low |  | 1769/6874 |  | 1582/13,729 | 283/2177 | 121/266 |
| Model 1 | |  |  |  |  |  |  |
|  | All |  | 1.00 |  | 0.89 (0.86, 0.92) | 0.82 (0.79, 0.86) | 0.93 (0.87, 0.99) |
|  | High |  | 1.00 |  | 0.87 (0.82, 0.93) | 0.80 (0.74, 0.87) | 0.86 (0.78, 0.96) |
|  | Middle |  | 1.00 |  | 0.92 (0.88, 0.96) | 0.88 (0.83, 0.94) | 0.97 (0.87, 1.08) |
|  | Low |  | 1.00 |  | 0.93 (0.86, 1.00) | 0.90 (0.79, 1.03) | 1.48 (1.23, 1.79) |
| Model 2 | |  |  |  |  |  |  |
|  | All |  | 1.00 |  | 0.93 (0.90, 0.96) | 0.91 (0.87, 0.95) | 1.01 (0.94, 1.08) |
|  | High |  | 1.00 |  | 0.90 (0.84, 0.95) | 0.86 (0.79, 0.93) | 0.93 (0.84, 1.04) |
|  | Middle |  | 1.00 |  | 0.94 (0.90, 0.98) | 0.94 (0.88, 1.00) | 0.96 (0.86, 1.06) |
|  | Low |  |  |  | 0.92 (0.86, 0.99) | 0.91 (0.80, 1.03) | 1.49 (1.24, 1.80) |
| Effect modification | |  |  |  |  |  |  |
|  | Middle vs high (ref) |  |  |  | 1.05 (0.98, 1.13), p=0.14 | 1.10 (0.99, 1.21), p=0.07 | 1.02 (0.88, 1.18), p=0.80 |
|  | Low vs high (ref) |  |  |  | 1.06 (0.97, 1.16), p=0.17 | 1.10 (0.95, 1.27), p=0.22 | 1.63 (1.32, 2.01), p=0.000001 |
|  | Low vs middle (ref) |  |  |  | 1.01 (0.93, 1.09), p=0.80 | 1.00 (0.87, 1.15), p=0.99 | 1.60 (1.29, 1.98), p=0.00001 |

Abbreviations: SEP=socioeconomic position. Hazard ratios (HRs) and 95% confidence intervals (CIs) derived from Cox models. HRs among current drinkers (ordinal) with infrequent consumers as reference category. Models (1) included age and gender, and (2) smoking, body mass index, diabetes, physical activity, history of cardiovascular disease, family history of coronary heart disease, systolic blood pressure, heart rate, triglycerides, and life course socioeconomic position (if not used as a stratifying variable). Effect modification (using model 2) was tested on multiplicative scale and used the high or middle SEP strata as reference category.
