# Supplementary material for: Life course socioeconomic position, alcohol drinking patterns in midlife, and cardiovascular mortality: Analysis of Norwegian population-based health surveys
Source: PLoS Med. 2018 Jan 2;15(1):e1002476. doi: 10.1371/journal.pmed.1002476 (PMC5749685; doi:10.1371/journal.pmed.1002476)
Supplement: S12 Table — (DOCX) [file pmed.1002476.s015.docx]

## **S12 Table.** Ischemic heart disease mortality according to binge drinking episodes in the subgroup with data on binge drinking (n=32,616).

| **Life course SEP** | |  |  |  | Current binge drinkers | | |
| --- | --- | --- | --- | --- | --- | --- | --- |
| N with/without event or HR (95% CI) for IHD mortality | |  | Not last year (n=15,657) |  | A few times last year (n=11,001) | 1-3 times per month (n=4729) | ≥1 time per week (n=1229) |
|  | All |  | 544/15,113 |  | 305/10,696 | 127/4602 | 52/1177 |
|  | High |  | 165/4827 |  | 101/3415 | 30/1608 | 15/440 |
|  | Middle |  | 285/8175 |  | 153/5818 | 79/2384 | 30/579 |
|  | Low |  | 94/2111 |  | 51/1463 | 18/610 | 7/158 |
| Model 1 | |  |  |  |  |  |  |
|  | All |  | 1.00 |  | 1.06 (0.91, 1.24) | 1.11 (0.90, 1.36) | 1.78 (1.33, 2.39) |
|  | High |  | 1.00 |  | 1.11 (0.85, 1.45) | 0.75 (0.50, 1.12) | 1.38 (0.80, 2.37) |
|  | Middle |  | 1.00 |  | 1.08 (0.88, 1.34) | 1.54 (1.18, 2.02) | 2.43 (1.64, 3.60) |
|  | Low |  | 1.00 |  | 0.96 (0.66, 1.40) | 0.80 (0.47, 1.38) | 1.27 (0.58, 2.80) |
| Model 2 | |  |  |  |  |  |  |
|  | All |  | 1.00 |  | 1.05 (0.90, 1.23) | 1.09 (0.88, 1.34) | 1.62 (1.20, 2.17) |
|  | High |  | 1.00 |  | 1.08 (0.82, 1.41) | 0.70 (0.47, 1.06) | 1.32 (0.76, 2.29) |
|  | Middle |  | 1.00 |  | 1.04 (0.84, 1.29) | 1.53 (1.16, 2.01) | 1.89 (1.27, 2.80) |
|  | Low |  | 1.00 |  | 1.01 (0.69, 1.48) | 0.74 (0.43, 1.29) | 1.24 (0.56, 2.76) |
| Effect modification | |  |  |  |  |  |  |
|  | Middle vs high (ref) |  |  |  | 0.91 (0.66, 1.24), p=0.54 | 2.03 (1.28, 3.22), p<0.01 | 1.42 (0.74, 2.73), p=0.29 |
|  | Low vs high (ref) |  |  |  | 1.06 (0.70, 1.62), p=0.77 | 1.28 (0.67, 2.41), p=0.45 | 1.17 (0.46, 2.98), p=0.74 |
|  | Low vs middle (ref) |  |  |  | 1.18 (0.79, 1.74), p=0.42 | 0.63 (0.36, 1.11), p=0.11 | 0.82 (0.35, 1.94), p=0.65 |
| Model 3 | |  |  |  |  |  |  |
|  | All |  | 1.00 |  | 1.14 (0.98, 1.34) | 1.32 (1.06, 1.66) | 2.17 (1.57, 3.01) |
|  | High |  | 1.00 |  | 1.23 (0.93, 1.63) | 0.94 (0.60, 1.45) | 2.05 (1.12, 3.73) |
|  | Middle |  | 1.00 |  | 1.13 (0.91, 1.41) | 1.89 (1.40, 2.56) | 2.57 (1.67, 3.98) |
|  | Low |  | 1.00 |  | 1.01 (0.68, 1.49) | 0.74 (0.41, 1.33) | 1.01 (0.42, 2.47) |

Abbreviations: IHD=ischemic heart disease; SEP=socioeconomic position. Hazard ratios (HRs) and 95% confidence intervals (CIs) derived from Cox models. Models (1) included age and gender, (2) smoking, body mass index, diabetes, physical activity, history of cardiovascular disease, family history of coronary heart disease, systolic blood pressure, heart rate, triglycerides and life course socioeconomic position (if not used as a stratifying variable), and (3) the frequency of alcohol consumption. Effect modification (using model 2) was tested on multiplicative scale and used the high or middle SEP strata as reference category.
